# Accelerated epigenetic age in hypertension: a systematic review and meta-analysis

**DOI:** 10.1038/s41440-025-02470-y

**Published:** 2026-01-09

**Authors:** C. Dollin, M. Ward, M. Y. C. Stafford, E. Krason-Kidzinska, Lauren Crawford, H. McNulty, Frank Barry, M. Murphy, D. J. Lees-Murdock

**Affiliations:** 1 Centre for Genomic Medicine, Biomedical Sciences Research Institute, Ulster University, Coleraine, N. Ireland UK; 2 Nutrition Innovation Centre for Food and Health (NICHE), Biomedical Sciences Research Institute. Ulster University, Coleraine, Northern Ireland; 3 Regenerative Medicine Institute (REMEDI), University of Galway, Galway, Ireland

**Keywords:** DNA methylation, hypertension, epigenetic Age, epigenetics, epigenomics, biological Age

## Abstract

Chronological age is a well-established risk factor for Hypertension (HTN), yet while biological ageing markers such as epigenetic age acceleration (EAA), have been associated with HTN, findings are inconsistent. This study aimed to conduct a systematic review and meta-analysis to evaluate the association between EAA, HTN and blood pressure (BP) to provide an understanding of the role of EAA in HTN development and progression. Six databases were searched, and studies which reported associations between DNA and HTN, and/or BP were included. Functional enrichment analysis was conducted using DAVID and STRING to elucidate underlying molecular pathways. From 4334 studies, 165 met the inclusion criteria. Qualitative analysis indicated that 17.0% of studies reporting global methylation and 49.1% of studies reporting gene-specific methylation demonstrated significant associations with HTN and/or BP. A random effects meta-analysis of 16,136 participants from 8 studies using three epigenetic clock algorithms demonstrated that HTN was associated with increased EAA (β: 0.29, 95%Cl: 0.15–0.43; *P* < 0.0001). All three individual epigenetic clocks demonstrated a positive association between clinically measured HTN and EAA (Horvath β: 0.33, 95%Cl: 0.08–0.58, *P* = 0.010; Hannum β: 0.64, 95%Cl: 0.09–1.20; PhenoAge β: 1.21, 95%Cl: 0.56–1.86), whereas this relationship was not clear when using self-reported HTN. This study is the first to systematically demonstrate that HTN is associated with EAA. We recommend the use of clinically measured over self-reported HTN in appropriately powered studies of epigenetic age to obtain an accurate understanding of BP regulation/HTN on the epigenome, supporting pathways to translation and development of novel therapeutic targets for HTN.

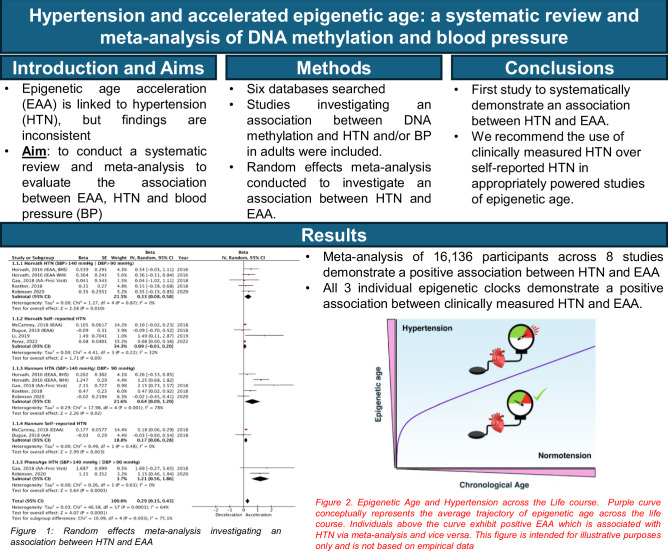

## Introduction

Hypertension (HTN) is the leading modifiable risk factor for cardiovascular disease (CVD), a highly prevalent worldwide contributor to mortality, responsible for 8.5 million deaths annually [1]. While advancements in diagnosis and treatment have been observed in high- and middle-income countries, HTN remains highly prevalent, affecting 31% of adults worldwide. From 1990 to 2019, the global prevalence of HTN has doubled and more than 50% of HTN cases remain undiagnosed [2, 3]. Despite the availability of medical and lifestyle interventions to improve cardiovascular outcomes, HTN control rates remain poor, with only 24% of women and 20% of men worldwide achieving target BP [3, 4].

Hundreds of common single-nucleotide polymorphisms (SNPs) have been identified for HTN, a heritable complex polygenic trait. Genetic variants, however, do not fully explain HTN heritability suggesting the involvement of additional mechanisms [5–7]. Epigenetic modifications, such as DNA methylation, have been implicated in HTN, BP and stroke [8–10]. DNA methylation is responsive to environmental factors, such as diet and nutritional status, and may mediate the interaction between genetic predisposition and development and progression of HTN [11–14].

Specific subsets of CpG sites undergo programmed methylation changes which have informed the development of biological clock algorithms to provide estimates of epigenetic age [15]. The difference between chronological and epigenetic age provides a measure of EAA where higher epigenetic age than chronological age indicates accelerated epigenetic age, and vice versa. Various epigenetic clocks, including the Horvath and Hannum algorithms, have been developed to predict epigenetic age and its association with disease states [16–18]. Second-generation clock algorithms, such as DNAmGrimAge and DNAmPhenoAge, incorporate age-related biomarkers to improve accuracy in assessing biological ageing and estimating all-cause mortality risk [18–20]. Recently, epigenetic clocks such as Horvath, Hannum and PhenoAge have demonstrated clinical utility, in patients with chronic kidney disease who exhibited increased EAA, an effect mitigated by transplantation but not dialysis [21].

Despite growing interest in this area, current evidence linking epigenetic age and BP or HTN remains conflicting. Several studies have reported significant associations between epigenetic clock algorithms, such as Horvath and HTN as well as systolic and diastolic BP [22, 23]. In contrast, other studies have reported no such associations [24, 25]. This inconsistency extends to studies investigating HTN and/or BP using more targeted DNA methylation assessment methods, including global, gene-specific and epigenome-wide approaches [26–29]. Consequently, despite increasing evidence supporting the involvement of DNA methylation in HTN [8, 10], there is are a lack of systematic analysis and evaluation of the existing evidence linking various measures of DNA methylation (global, gene-specific, epigenome-wide), epigenetic age and HTN.

The aim of this study was to conduct a systematic review to investigate the association between DNA methylation and HTN and/or BP in adults. Furthermore, we performed a comprehensive meta-analysis of studies in adults reporting epigenetic age performed using various clock algorithms to determine the association between HTN and EAA. Finally, functional enrichment analysis was conducted to explore the relationship between epigenome-wide DNA methylation and biological pathways in HTN.

## Methods

This review was conducted in accordance with the Preferred Reporting Items for Systematic Reviews and Meta-analyses (PRISMA) guidelines. Screening of eligible studies, full-text assessment, data extraction, and quality assessment of studies were independently carried out by two authors. Any discrepancies were discussed and resolved by consensus. Studies were selected in line with the PICOS (Population, Intervention, Comparison, Outcomes, Study Type) criteria outlined in Table S1.

### Search strategy and study selection

Systematic searches were performed in 6 bibliographic databases (Medline (Ovid), Embase, Cumulative Index to Nursing and Allied Health Literature (CINAHL), Cochrane Library, Web of Science and Scopus). The search covered the period from January 1^st^ 2000 to 14^th^ October 2024 (date of last search). The search strategy combined terms related to DNA methylation (e.g., global, gene-specific, epigenome-wide) and BP (e.g., systolic, diastolic, HTN, high BP). Medical subject headings and keyword searches were conducted in databases where a thesaurus was available (Embase, Medline, CINAHL, Cochrane Library), while keyword searches were performed in other databases (Web of Science, Scopus). Only full text studies involving humans, published in English were included. Detailed search terms and search strategies are provided in Table S2. Retrieved records from databases were exported to the systematic review manager Rayyan [30] for the removal of duplicates. Full-text studies selected for inclusion were further imported into an additional systematic review manager, Covidence (www.covidence.org), where studies were further assessed for full-text eligibility and data extraction.

### Inclusion and exclusion criteria

Studies were deemed eligible for inclusion in this review if they were original, peer-reviewed, full-text articles published in English and met all defined inclusion criteria. These criteria included studies that: (1) assessed DNA methylation, specifically 5-methylcytosine (5mC) (global, gene-specific and epigenome-wide); (2) measured or recorded data on HTN and/or BP; (3) were conducted in adult humans aged >18 years; and (4) investigated an association between DNA methylation and HTN and/or BP. HTN status was defined to the European Society of Hypertension/European Society of Cardiology guidelines (HTN defined as systolic blood pressure (SBP) ≥140 mmHg and/or diastolic blood pressure (DBP) ≥90 mmHg and/or anti-HTN usage) [31] and the American College of Cardiology/American Heart Association Task Force guidelines (HTN defined as SBP ≥ 130 mmHg and/or DBP ≥ 80 mmHg and/or anti-HTN usage [32]. Additionally, we included studies that defined HTN as self-reported and studies which provided a diagnosis of HTN from medical history. No restrictions were imposed regarding the tissue in which DNA methylation was assessed allowing for a comprehensive analysis of the current landscape of studies. Animal studies, studies involving pregnant women and children, or in vitro studies using human or animal cell lines were excluded from this review (Table S1).

### Risk of bias assessment

Bias within each included study was assessed using the Newcastle-Ottawa Scale [33] a semi-quantitative scale designed to evaluate the quality of non-randomised studies. Study quality was assessed based on the selection criteria of participants, comparability and exposure and outcome assessment. Studies that received a score of 9 stars were considered to have a low risk of bias, and those that scored 7–8 stars were considered to have a medium risk of bias; and those that scored less than 7 were considered to have a high risk of bias.

### Data extraction and analysis

A predesigned data collection form was created within *Covidence* to extract the relevant information from the included studies, including the first author, study design, percentage of male participants, age range, sample size and location where possible. Additionally, a description of the cohorts, such as the names of large prospective cohorts or the diseased population (e.g., patients with HTN), was included. Furthermore, information regarding the type of tissue, molecular technique and outcomes related to DNA methylation was extracted.

A qualitative analysis was presented for associations between DNA methylation (global methylation, gene-specific, and epigenome-wide) and HTN and/or BP, however, due to considerable heterogeneity in study aims, meta-analysis was not appropriate for these measures. Instead, a meta-analysis was conducted to examine the association between HTN and EAA which employed 3 different epigenetic clock algorithms; Horvath, Hannum and PhenoAge.

### Meta-analysis

We conducted a random-effects meta-analysis to examine the effect of HTN on EAA. The meta-analysis included studies that investigated the association between HTN and EAA, where EAA was reported as an outcome. Studies eligible for inclusion in the meta-analysis measured at least one of the definitions of EAA. Only studies reporting HTN were included rather than other measures of BP due to the low number of studies reporting statistically comparable outcomes for BP. All eligible studies for meta-analysis, defined HTN as SBP > 140 or DBP > 90 and/or use of antihypertensive medication or separately as self-reported HTN.

Subgroup analysis was performed to determine the effect of HTN on each epigenetic clock algorithm, both independently and as a combined effect of the 3 algorithms. While first-generation epigenetic clocks are derived by a linear regression algorithm that trains chronological age against a select set of CpGs [16–18] more recent clocks have included additional parameters, such as the inclusion of 9 biomarkers in PhenoAge [18]. Studies using GrimAge [19] were ineligible for inclusion in meta-analysis due to incompatible statistical reporting, such as reporting EAA as a predictor variable, which limited comparability with other studies included for meta-analysis. Furthermore to account for variability in HTN definitions, subgroup analysis was utilised to examine the association between HTN and EAA based on whether HTN was defined by European clinical guidelines [31] or by self-report.

### Statistical analysis

Review Manager Version 5.3 software was used to perform a random-effects meta-analysis [34]. We employed a random-effects model to account for expected heterogeneity in effect sizes across clocks and studies. The random-effects model estimates between-study variance, allowing for the assignment of weight to individual studies when calculating an overall pooled effect that reflects this variability. Beta effect estimations and standard errors were extracted from included studies that investigated epigenetic age as an outcome. In studies where standard errors were absent, the standard error was estimated from 95% confidence intervals using Cochrane formulas. Results were expressed as beta effect estimates and 95% confidence intervals, in addition to the overall effect Z value. Pre-specified subgroup analysis, grouped by epigenetic clock and by clinically measured vs self-reported HTN was performed.

Publication bias for studies included in the meta-analysis was assessed through visual inspection of funnel plots, Egger’s regression test and the trim-and-fill procedure using the *metafor* package within the statistical software platform R (Version 4.1.2) [35, 36].

Heterogeneity was assessed using chi-squared testing (χ value), heterogeneity index (I^2^) statistics and corresponding P value. Heterogeneity thresholds were predefined according to Cochrane guidelines, which stated that an I^2^ value between 0% and 40% indicates low heterogeneity, between 30% and 60% represents moderate heterogeneity, between 50% and 90% represents substantial heterogeneity, and between 75% and 100% indicates considerable heterogeneity. Sensitivity analysis was performed by conducting the meta-analysis excluding one study at a time to determine stability of the overall pooled effect across the 3 clock algorithms.

### Functional analysis

Functional analysis was conducted separately for previously identified CpG sites associated with BP and/or HTN within epigenome-wide association studies using the DAVID Bioinformatics Resource and the Search Tool for the Retrieval of Interacting Genes/Proteins (STRING), which allowed for the formation of protein-protein interaction networks [37, 38]. Clustering within STRING analysis was performed using the Markov Clustering Algorithm. Overlapping differentially methylated regions (DMR) were determined using the *GenomicRanges* package (Version 1.46.1) with R (Version 4.1.2) to identify DMRs with the same genomic region prior to visualisation. CpG sites and DMRs associated with BP traits were visualised individually using the InteractiVenn software [39].

## Results

**Fig. 1:**
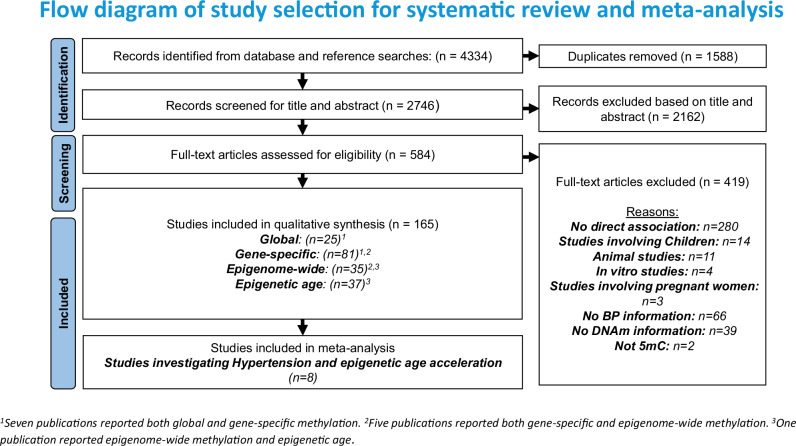
Flow diagram of study selection for systematic review and meta-analysis. ^a^Seven publications reported both global and gene-specific methylation. ^b^Five publications reported both gene-specific and epigenome-wide methylation. ^c^One publication reported epigenome-wide methylation and epigenetic age

Overall, 4334 potentially relevant records were identified through a systematic search of 6 databases. Based on the title and abstract, 584 articles were identified for detailed evaluation. After full-text assessment, 165 studies met the predefined eligibility criteria and were included in this review. Detailed screening, eligibility and selection processes are outlined in Fig. 1.

### Characteristics of included studies

**Fig. 2:**
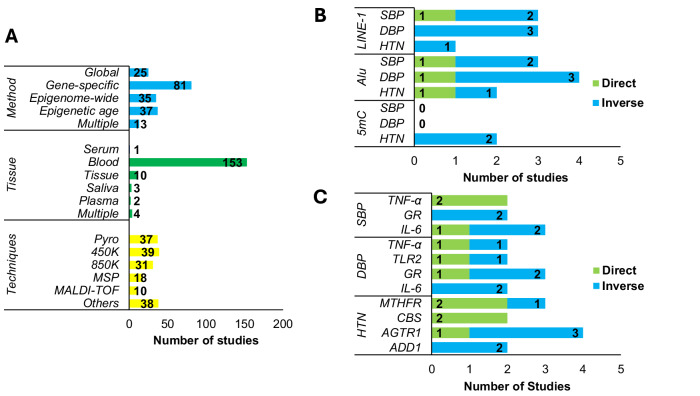
**A** Study characteristics stratified by methylation assessment method, tissue and technique (**B**) Studies reporting significant associations between BP traits and global DNA methylation stratified by global DNA methylation marker; (**C**) Assessment of studies reporting significant associations between BP traits and gene-specific DNA methylation

Overall, 165 studies were included that involved adults aged 18–99 years, ranging in size from 6 to 17,010 participants. Individuals were recruited from 34 different countries, with the most prevalent being China (29.1%), the USA (24.2%) and Spain (6.1%). DNA methylation was assessed in a gene-specific manner in 45.8% of studies, while 20.9% investigated epigenetic age, 19.8% of studies investigated epigenome-wide methylation, and 14.1% investigated global methylation (Fig. 2A). DNA methylation was predominantly examined in blood (91.1% of studies), followed by tissue (7.1%), saliva (1.8%), plasma (1.2%), and serum (0.6%). DNA methylation was examined in multiple tissues within 2.4% of studies. A total of 24 different techniques were used for DNA methylation analysis, with the majority employing Infinium HumanMethylation 450 K BeadChip microarray (Illumina, San Diego, CA) (23.6%), pyrosequencing (22.4%), Infinium MethylationEPIC BeadChip microarray (Illumina, San Diego, CA) (18.8%) and methylation-specific PCR (10.9%). Seven studies reported the use of more than one method. Detailed participant characteristics for each of the 165 studies within this review were stratified by global methylation (Table 1), gene-specific methylation (Table 2), epigenome-wide methylation (Table 3) and epigenetic age (Table 4).

**Table 1:** Associations between global DNA methylation and hypertension and/or blood pressure

Author, Year	Quality^1^	Population	Male (%), age (SD), sample size, country	Measure of Global Methylation	Method	Definition of HTN/BP	Tissue type	Associations between global methylation and HTN and/or blood pressure (BP).
Alexeeff et al. 2013 [26]	7/9	NAS	100, 74.1 (6.7), 789, USA	*LINE-1, Alu*	Pyro	HTN: SBP ≥ 140 mmHg and/or DBP ≥ 90 mmHg and/or anti-HTN usage. BP: seated BP was measured in both left and right arm.	WB	Inverse association between DBP (β = −0.70, 95%Cl: −1.20, -0.20) and *LINE-1*. Positive association between SBP (β = 1.40, 95%Cl: 0.57, 2.23), DBP (β = 0.81, 95%Cl: 0.36, 1.26) and *Alu*.
Amenyah et al. 2020 [11]	7/9	HTN	60, 56.8 (6.9), 80, N. Ireland	*LINE-1*	Pyro	HTN: SBP ≥ 140 mmHg and/or DBP ≥ 90 mmHg and/or anti-HTN usage. BP: 2 measurements taken 15 min apart seated after 15 min rest.	PBC	Baseline: At baseline *MTHFR* 677TT participants had higher BP than CC (*P* = 0.024) and higher *LINE-1* methylation (0.011). Intervention: Intervention by riboflavin resulted in significantly reduced BP specifically in *MTHFR* 677TT participants. *LINE-1* methylation was hypomethylated in TTs following intervention induced BP reduction (*P* = 0.018).
Baccarelli et al. 2010 [45]	6/9	NAS	100, 72.3 (n/a)^2^, 712, USA	*LINE-1*	Pyro	HTN: SBP ≥ 140 mmHg and/or DBP ≥ 90 mmHg and/or anti-HTN usage.	WB	Inverse association between HTN and *LINE-1* (OR = 0.6, 95%Cl: 0.3, 1.0) at baseline for subjects in the lowest vs highest quartiles of *LINE-1* methylation.
Bellavia et al. 2013 [51]	5/9	Healthy	53.3, 27.7 (8.6), 15, Canada	*LINE-1, Alu*	Pyro	HTN: SBP ≥ 140 mmHg and/or DBP ≥ 90 mmHg and/or anti-HTN usage. BP: seated BP was measured after 10 minutes rest.	WB	Decreased *Alu* methylation was associated with significantly increased DBP (β = 0.41, *P* = 0.04).
Burghardt et al. 2012 [106, 107]	6/9	Schizophrenia	48.1, 46.1 (11.1), 133, USA	*LINE-1*	Pyro	BP: indication of measurement.	WB	No association.
Carraro et al. 2016 [52]	7/9	Healthy	21, 28.9 (7.0), 40, Brazil	*LINE-1*	MS RT-PCR	BP: according to WHO guidelines.	PBC	No association.
Castellano-Castillo et al. 2019 [108]	6/9	MetS	44, 52.7 (14.6), 108, Spain	*LINE-1*	Pyro	HTN: SBP ≥ 130 mmHg and/or DBP mmHg ≥85 and/or anti-HTN usage.	VAT	No association.
Chen et al. 2014 [109]	6/9	APA	64, 48 (11), 25, China	*LINE-1*	MS RT-PCR	HTN: medical history.	APA	No association.
Childebayeva et al. 2019 [110]	6/9	Healthy	54.55, 24.1 (8.2), 21, Nepal	*LINE-1*	Pyro	BP: indication of measurement.	WB	No association.
Deng et al. 2018 [111]	6/9	CAD	57, 58.0 (9.5), 535, China	5mC	LC-MS	BP: medical history	PBC	No association.
Ferrari et al. 2019 [42]	7/9	HTN	73.5, 49.5 (22-70)^2^, 68, Italy	*LINE-1, Alu*	Pyro	BP: indication of measurement.	WB	SBP was inversely associated with *LINE-1* methylation (β = -0.90, P = 0.023).
Hossain et al. 2017 [43]	7/9	Arsenic-endemic	53.0, 35.7 (10.1), 236, Bangladesh	*LINE-1*	Pyro	BP: measured 3 times while sitting with the average taken. HTN: SBP ≥ 140 mmHg and/or DBP ≥ 90 mmHg and/or anti-HTN usage	WB	Negative association between SBP (β = −1.381, 95%Cl: −2.395, −0.366, *P* < 0.01), DBP (β = −0.987, 95%Cl: −1.669, −0.305) and *LINE-1*.
Jiraboonsri et al. 2023 [48]	4/9	Gen Pop	12.8, N/A^2^, 39, Thailand	*Alu*	COBRA-Alu MSP	HTN: SBP ≥ 140 mmHg and/or DBP ≥ 90 mmHg and/or anti-HTN usage	DT	Higher *Alu* methylation was observed in pre-HTN compared to NT (*P* < 0.05).
Kim et al. 2010 [112]	7/9	Singapore Chinese Health Study	55.6, 45-74^2^, 286, Singapore	*Alu* and *Satellite 2* repetitive element	MethylLight qPCR	HTN: self-reported.	PBC	No association.
Luttmer et al. 2013 [113]	6/9	Hoorn Screening Study	50.5, 68.7 (7.2), 738, Netherlands	MC/C-ratio	LC-MS	HTN: SBP ≥ 140 mmHg and/or DBP ≥ 90 mmHg and/or anti-HTN usage.	PBC	No association.
Maghbooli et al. 2014 [114]	5/9	T2DM	66.7, 58.3 (0.9), 123, Iran	5mC	RP-HPLC	BP: measured twice after 10 minutes rest. HTN: SBP ≥ 140 mmHg and/or DBP ≥ 90 mmHg and/or anti-HTN usage	PBC	No association.
Maghbooli et al. 2015 [115]	5/9	Diabetic retinopathy	55.45, 59.43 (0.7), 168, Iran	5mC	RP-HPLC	BP: measured twice after 10 minutes rest. HTN: SBP ≥ 140 mmHg and/or DBP ≥ 90 mmHg and/or anti-HTN usage.	PBC	No association.
Marques-Rocha et al. 2016 [116]	5/9	Healthy	41.7, 23.2 (3.5), 156, Brazil	*LINE-1*	MS-HRM	BP: measured to WHO criteria.	WBC	SBP was associated with highest quartile of *LINE-1* methylation (*P* = 0.038).
Smolarek et al. 2010 [49]	5/9	HTN	65, 38.4 (13.0), 60, Poland	5mC	TLC chromatography	HTN: SBP ≥ 140 mmHg and/or DBP ≥ 90 mmHg and/or anti-HTN usage	WB	Lower levels of 5mC in in individuals with stage 1 and 2 HTN vs controls (*P* < 0.0001).
Thongsroy et al. 2017 [46]	6/9	DM	30, 59.99 (11.9), 240, Thailand	*Alu*	COBRA-Alu	BP: indication of measurement.	BC	Negative association between SBP (r^2^ = −0.35, *P* = 0.0012), DBP (r^2^ = −0.31, *P* = 0.0045) and *Alu* in DM patients.
Thongsroy et al. 2022 [47]	6/9	HTN	20.4, 62.0 (11.8), 240, Thailand	*Alu*	COBRA-Alu	HTN: indication of measurement.	BC	Lower methylation in HTN vs NT (*P* = 0.0002). Inverse correlation between SBP (r = −0.272, *P* = 0.047), DBP (r = −0.628, *P* < 0.0001) and *Alu*.
Turcot et al. 2012 [44]	6/9	Obesity	22.7, 35.3 (7.3), 186, Canada	*LINE-1*	Pyro	BP: measured sitting after 5 minutes rest. HTN: SBP ≥ 130 mmHg and/or DBP ≥ 80 mmHg and/or antihypertensive usage	VAT	DBP was negatively associated with *LINE-1* methylation (β = −0.65, 95%Cl: −1.26, −0.05, *P* = 0.03).
Wei et al. 2014 [117]	6/9	CHD	81, <59: 33.8, 60–65: 29.9, >65: 36.3^2^, 334, China	*LINE-1*	Pyro	BP: medical history	PBC	No association.
Wu et al. 2017 [118]	6/9	T2DM	50.7, 61.2 (10.5), 418, China	*LINE-1*	qMSP	BP: indication of measurement.	WB	No association.
Yadav et al. 2021 [50]	6/9	HTN	38.1, 50 (42–57)^2^, 1634, North India	5mC	MethylFlash Methylated DNA 5-mC ELIZA	HTN: SBP ≥ 140 mmHg and/or DBP ≥ 90 mmHg and/or anti-HTN usage	WB	Negative association between untreated HTN and 5mC (r^2^ = −0.859, *P* = 0.01).

*APA* aldosterone producing adenoma, *BC* buffy coat, *BP* Blood pressure, *CAD* Coronary artery disease, *CHD* coronary heart disease, *COBRA-Alu* Alu-Combined Bisulfite Restriction Analysis, *DBP* Diastolic blood pressure, *DM* diabetes mellitus, *DT* Dermal Tissue, *ELIZA* enzyme linked immunosorbent assay, Gen pop General population, *HTN* hypertension, *MetS* metabolic syndrome, *MS-HRM* methylation sensitive high resolution melt curve analysis, *MS RT-PCR* methylation specific reverse-transcription polymerase chain reaction, *LC-MS* liquid chromatography mass spectroscopy, *NAS* normative aging study, *NT* normotensive, *PBC* peripheral blood cells, *Pyro* pyrosequencing, *qMSP* quantitative methylation specific polymerase chain reaction, *qPCR* quantitative polymerase chain reaction, *RP-HPLC* Reversed phase-high pressure liquid chromatography analysis, *SBP* systolic blood pressure, *T2DM* type 2 diabetes mellitus, *TLC* chromatography, Thin-layer chromatography, *VAT* visceral adipose tissue, *WB* whole blood, *WBC* white blood cells;

^1^Quality assessment determined using Newcastle-Ottawa scale Highest score 9/9

^2^Age is either given as median (IQR), SD is unavailable, or age is not described

**Table 2:** Associations between blood pressure or hypertension and gene-specific DNA methylation

Author, Year	Quality^1^	Population	Male (%), age (SD), sample size, country	Methylation sites	Method	Definition of HTN/BP	Tissue type	Associations between gene-specific methylation and HTN and/or BP
Abula et al. [119]	5/9	HTN and MDD	65.6, 57.4 (9.7), 208, China	*ACE*	MSP	HTN: SBP ≥ 140 mmHg and/or DBP ≥ 90 mmHg and/or anti-HTN usage.	Serum	Positive correlations were identified between the DNA methylation level of *ACE* and SBP (r = 0.4635, P < 0.0001) and DBP (r = 0.3095, P = 0.0006).
Alexeeff et al. 2013 [26]	7/9	NAS	100, 74.1 (6.7), 789, USA	*TLR2, iNOS, IFNγ, F3, GCR*, *ICAM-1*	Pyro	HTN: SBP ≥ 140 mmHg and/or DBP ≥ 90 mmHg and/or anti-HTN usage. BP: seated BP was measured in both left and right arm.	WB	Positive association between DBP and *iNOS* (β = 0.91, 95%Cl: 0.24, 1.57) and *TLR2* (β = 1.16, 95%Cl: 0.53, 1.78). Negative association between DBP and *IFNy* (β = -1.10, 95%Cl: -1.68, -0.52). Negative association between SBP and *IFNy* (β = -1.70, 95%Cl: -2.76, -0.63).
Ali et al. 2021 [120]	5/9	Obesity	50, 36.2 (1.6), 48, USA	*ATF2, BCL10, CCL25, CSF1, CXCL1, CXCL12, CXCL2, CXCL6, CXCR4, EGR1, FADD, HDAC4, IGF2BP2, IL12A, IL5RA, IL17RA, IL6R, IL6ST, IL17, JUN, TNFRSF1B, TRAF2, TLR5*,	EpiTect Methyl II PCR Array	BP: indication of measurement.	WB	Negative correlation between SBP and *ATF2* (β = -0.453, P = 0.001), *BCL10* (β = -0.346, P = 0.008)*, CCL25* (β = -0.385, P = 0.003)*, CSF1* (β = -0.387, P = 0.003)*, CXCL1* (β = -0.371, P = 0.005)*, CXCL12* (β = -0.270, P = 0.032)*, CXCL2* (β = -0.312, P = 0.015)*, CXCL6* (β = -0.420, P = 0.001)*, CXCR4* (β = -0.294, P = 0.021)*, EGR1* (β = -0.330, P = 0.011)*, FADD* (β = -0.271, P = 0.031)*, HDAC4* (β = -0.382, P = 0.004)*, IGF2BP2* (β = -0.404, P = 0.002)*, IL12A* (β = -0.393, P = 0.003)*, IL5RA* (β = -0.378, P = 0.004)*, IL17RA* (β = -0.418, P = 0.002), *IL6R* (β = -0.420, P = 0.001)*, IL6ST* (β = -0.332, P = 0.011)*, IL7* (β = -0.325, P = 0.012)*, JUN* (β = -0.386, P = 0.003)*, TLR5* (β = -0.380, P = 0.004)*, TNFRSF1B* (β = -0.396, P = 0.003)*, TRAF2* (β = -0.429, P = 0.001). Negative correlation between DBP and *BCL10* (β = -0.324, P = 0.012)*, CCL25* (β = -0.320, P = 0.013)*, CSF1* (β = -0.325, P = 0.012)*, CXCL2* (β = -0.294, P = 0.021)*, CXCR4* (β = -0.280, P = 0.027), *EGR1* (β = -0.277, P = 0.028)*, IL12A* (β = -0.249, P = 0.044)*, IL6R* (β = -0.354, P = 0.007)*, IL6ST* (β = -0.306, P = 0.017)*, JUN* (β = -0.244, P = 0.047)*, TRAF2* (β = -0.247, P = 0.045).
Amenyah et al. 2021 [14]	7/9	HTN	60, 56.8 (6.9), 80, N. Ireland	*ACE, AGTR1, GCK, GNA12, IGF2, MMP9, NOS3*	Pyro	HTN: SBP ≥ 140 mmHg and/or DBP ≥ 90 mmHg and/or anti-HTN usage. BP: 2 measurements taken 15 minutes apart seated after 15 minutes rest.	PBC	Baseline: At baseline *MTHFR* 677TT participants had higher BP than CC (P = 0.024) and higher average NOS3 methylation (P = 0.044) and *AGTR1* (P = 0.048) and *GNA12* (P = 0.006) methylation at individual CpG sites. Intervention: Intervention by riboflavin resulted in significant BP reduction specifically in *MTHFR* 677TT participants. *IGF2* and *AGTR1* were hypermethylated at 1 CpG site, while *GNA12* was hypomethylated at 2 CpGs sites in TT participants following intervention induced BP reduction. *ACE* was hypomethylated in female TT participants.
Amenyah et al. 2020 [11]	7/9	HTN	60, 56.8 (6.9), 80, N. Ireland	*MTHFR*	Pyro	HTN: SBP ≥ 140 mmHg and/or DBP ≥ 90 mmHg and/or anti-HTN usage. BP: 2 measurements taken 15 minutes apart seated after 15 minutes rest.	PBC	Baseline: At baseline *MTHFR* 677TT participants had higher BP than CC (P = 0.024) and higher *MTHFR* south shelf and CpG island methylation at 1 CpG site. Intervention: Intervention by riboflavin resulted in significant BP reduction specifically in *MTHFR* 677TT participants. Lower *MTHFR* north shore methylation was associated with intervention induced BP reduction in TT participants (P = 0.001).
Baccarelli et al. 2015 [121]	4/9	CVD	30, 62.4 (n/a)^2^, 10, USA	*MT-CO1, MT-CO2, MT-CO3, MT-TL1, MT-ATP6, MT-ATP8, MT-ND5*	Pyro	HTN: medical history.	Plasma	Higher methylation in *MT-CO1, MT-CO2, MT-CO3* and *MT-TL1* within HTN individuals vs NTs (all P < 0.0001). No association with *MT-ATP6, MT-ATP8* and *MT-ND5*.
Bai et al. 2022 [122]	7/9	HTN	Discovery: 1:1 Ratio, 58.2 (5.1), 132, China Validation: 1:0.8 ratio, 59.96 (5.5), 203, China	*OVGP1, MAML2 PSORS1C1, ATP9B* *CPO, SLC37A1* *KCNK17, KRT82* *TLE4*	Pyro	HTN: Double-arm SBP and DBP values ≥ 160 mmHg and ≥110 mmHg respectively, or SBP ≥ 140 mmHg and/or DBP ≥ 90 mmHg after receiving anti-HTN treatment.	PBC	Discovery: Lower methylation in HTN vs NT individuals in *OVGP1* (P = 0.048), *CPO* (P = 0.015), *SLC37A1* (P = 0.043), *KCNK17* (P = 0.009) and *KRT82* (P = 0.037). Validation: Lower *OVGP1* methylation in HTN vs NT individuals (P = 0.003).
Bao et al. 2018 [123]	7/9	HTN	39.5, 56.7 (8.7), 96, China	*IFNG*	Pyro	HTN: SBP ≥ 140 mmHg and/or DBP ≥ 90 mmHg and/or anti-HTN usage.	PBC	Higher methylation observed in HTN vs NT at 2 CpGs (P = 0.003 and P = 5.87E-07). Lower methylation observed in HTN vs NT at 1 CpG (P = 1.24E-12). After logistic regression analysis 1 CpG was positively associated with HTN (P = 0.032).
Bayoumy et al. 2017 [61]	5/9	HTN	46.7, 53.2 (5.1), 150, Egypt	*ADD1*	Pyro	HTN: SBP ≥ 140 mmHg and/or DBP ≥ 90 mmHg and/or anti-HTN usage.	WB	Lower methylation observed in HTN vs NT at CpG1 (P = 0.021). Lower methylation in male HTN vs NT at CpG2-5 (P = 0.003). Lower methylation in female HTN vs NT in CpG1 (P = 0.003).
Bellavia et al. 2013 [51]	5/9	Healthy	53.3, 27.7 (8.6), 15, Canada	*TLR4, IL-6, IL-12, iNOS*	Pyro	BP: seated BP was measured after 10 minutes rest.	WB	Inverse association between *TLR4* methylation and DBP (P = 0.02) and SBP (P = 0.01).
Bi et al. 2024 [124]	7/9	CHD	50.6, 66.6 (60.3, 71.4), 666, China	*HYAL2*	MALDI-TOF	HTN: self-reported	PBC	Positive correlation between CpG1 (P = 0.03), CpG3 (P = 0.001) and CpG4 (P = 0.03) and HTN in controls. In CHD cases v controls CpG2 was associated with HTN (OR per − 10% methylation (95% CI) = 1.60 (1.15–2.23), p = 0.005)
Breitling et al. 2012 [125]	5/9	KAROLA	84.5, 30-70^b^, 1100, Germany	*F2RL3*	MALDI-TOF	HTN and BP: medical history.	WB	Higher methylation at CpG4 within HTN vs NTs (all P < 0.001).
Bushueva et al. 2021 [126]	5/9	Cerebral stroke	54.2, 57 (51.5-63.5)^2^, 59, Russia	*MPO, GCLM*	Pyro	HTN: medical history and anti-HTN usage.	WB	Lower *GCLM* methylation at 1 CpG in HTN vs NT controls (P < 0.05). Lower *MPO* methylation in HTN vs NT (P < 0.05).
Carraro et al. 2016 [127]	6/9	Healthy	0, 20.7 (2.3), 40, Spain	*TNF-α*	MALDI-TOF	BP: measured to WHO criteria.	WBC	Positive correlation between *TNF-α* and SBP (r = 0.32, P = 0.042).
Carraro et al. 2016 [52]	7/9	Healthy	21, 28.9 (7.0), 40, Brazil	*IL-6*	MS RT-PCR	BP: measured to WHO criteria.	PBC	Positive correlation between *IL-6* and SBP (r = 0.462, P = 0.003).
Castellano-Castillo et al. 2018 [128]	6/9	MetS	40, 49.8 (15.0), 134, Spain	*LPL*	Pyro	HTN: SBP ≥ 130 and/or DBP ≥ 85 and/or anti-HTN usage.	VAT	No association
Castellano-Castillo et al. 2019 [108]	6/9	MetS	44, 52.7 (14.6), 108, Spain	*PPARA, RXRA, SREBF1, SREBF2, SCD, LPL, LRP1, C3, LEP, TNF, PPARG*	Pyro	HTN: SBP ≥ 130 and/or DBP ≥ 85 and/or anti-HTN usage. BP: indication of measurement	VAT	DBP was negatively correlated with *PPARG P1* (r = -0.293, P < 0.05), *PPARG P3* (r = -0.283, P < 0.05), *SREBF2 P2* (r = -0.262, P < 0.05) and *TNF P4* (r = -0.305, P < 0.05). DBP was positively correlated with *LEP P1* (r = 0.230, P < 0.05).
Chen et al. 2020 [129]	6/9	OSA	82, 47.1 (8.0), 78, Taiwan	*FPR1, FPR2, FPR3*	Pyro	HTN: medical history.	PBC	*FPR2* methylation at 2 CpG sites was higher in HT vs NT (P = 0.013, P = 0.049).
Chen et al. 2023 [130]	7/9	TWB	43.4, 49.1 (0.3), 1357	*GPNMB*	850k	HTN: SBP ≥ 140 mmHg and/or DBP ≥ 90 mmHg and/or self-reported history of HTN.	WB	Higher cg17274742 methylation in HTN vs non-HTN (β = 0.002, P = 0.017).
Childebayeva et al. 2019 [110]	6/9	Healthy	54.55, 24.1 (8.2), 21, Nepal	*EPO, EPAS1, PPARa, RXRa*	Pyro	BP: indication of measurement.	WB	Positive association between *EPO* and SBP (β = 0.63, P = 0.022).
Chiu et al. 2023 [63]	7/9	TWB	49.1, 50.9 (10.8), 1278, Taiwan	*MTHFR*	850 K	HTN: physician diagnosed.	WB	Lower *MTHFR* promoter methylation quartiles associated with HTN in CC individuals (P < 0.05).
Cordero et al. 2011 [131]	5/9	Obesity	0, 32-50^2^, 27, Spain	*TNF-α*	MSP	BP: indication of measurement.	SAT	*TNF-α* was positively correlated with SBP (r = 0.421, P = 0.029) and DBP (r = 0.471, P = 0.013).
Corsi et al. 2020 [132]	7/9	SPHERE	39, 62.5 (10), 200, Italy	*MT-CO1, MT-CO3, MT- TL1*	Pyro	BP: measured supine after 5 minutes rest.	Plasma	Negative association between *MT-CO3* and SBP (β = -0.02, P = 0.017). Positive association between MT-CO3 and DBP (β = 0.012, P = 0.009).
Cortese et al. 2016 [133]	5/9	OSA	40, 50.9 (10.7), 15, USA	*ABCA1, ABCG1, CD36, FABP4, HMOX, NOS2, PEPCK, ADIPOQ*	MeDIP-qPCR	HTN: existing diagnosis.	CD14+	Higher *FABP4* methylation in HTN vs NT (P = 0.025).
Diniz et al. 2021 [134]	5/9	T2DM	39, 58.2 (9.4), 111, Brazil	*MTHFR*	MSP	BP: indication of measurement.	WB	No association.
Drake et al. 2012 [135]	7/9	Healthy	35.3, 40.0 (0.6), 34, Scotland	*11*β*HSD2, GR, H19*	Pyro	BP: indication of measurement.	BC	Positive association between SBP (β = 0.54, P = 0.002), DBP (β = 0.44, P = 0.03) and *H19* ICR. Inverse correlation between SBP (r = -0.40, P = 0.037), DBP (r = -0.37, P = 0.046) and *GR*. Positive correlation between SBP and 3 CpGs of *11*β*HSD2* (r = 0.42, P < 0.05; r = 0.43, P < 0.05; r = 0.50, P < 0.05; r = 0.45, P < 0.05; r = 0.43, P < 0.05). Negative correlation between SBP and 1 CpG of *11*β*HSD2* (r = -0.37, P < 0.05).
El Alami et al. 2022 [136]	5/9	T2DM	21.5, 55.1 (11.7), 209, Morocco	*MCP-1*	MSP	BP: indication of measurement.	PBC	No association.
Fan et al. 2015a [137]	6/9	HTN	N/A^3^, 59.2 (7.6), 94, China	*GCK*	Pyro	HTN: SBP ≥ 140 mmHg and/or DBP ≥ 90 mmHg and/or anti-HTN usage.	PBC	Lower *GCK* methylation at CpG1-3 in HTN vs NT (P = 0.006). Higher *GCK* methylation at 1 CpG HTN vs NT (P = 0.002).
Fan et al. 2015b [56]	7/9	HTN	39.5, 56.7 (8.7), 192, China	*AGTR1*	Pyro	HTN: SBP ≥ 140 mmHg and/or DBP ≥ 90 mmHg and/or anti-HTN usage.	PBC	Lower *AGTR1* methylation at 1 CpG in HTN vs NT (P = 0.007).
Fan et al. 2017 [138]	7/9	HTN	60.4, 56.7 (8.7), 192, China	*ACE2*	Pyro	HTN: SBP ≥ 140 mmHg and/or DBP ≥ 90 mmHg and/or anti-HTN usage.	PBC	Lower *ACE2* methylation at 2 CpGs in HTN vs NT (P = 0.02, P = 0.036).
Ferrari et al. 2019 [42]	7/9	HTN	73.5, 49.5 (22–70)^2^, 68, Italy	*NOS2, NOS3, ICAM1, TLR2, EDN1, TNF*	Pyro	BP: indication of measurement.	WB	*EDN1* methylation was inversely associated with SBP (β = -3.0, P = 0.003) and DBP (β = -1.7, P = 0.035). *NOS3* methylation was inversely associated with SBP (β = -0.4, P = 0.004) and DBP (β = -0.3, P = 0.008).
Friso et al. 2008 [139]	5/9	HTN	56, 51.9 (14.3), 57, Italy	*11*β*HSD2*	MSP	HTN: SBP ≥ 140 mmHg and/or DBP ≥ 90 mmHg and/or anti-HTN usage.	PBMC	Higher *11*β*HSD2* methylation in HTN vs NT (P = 0.018).
Gao et al. 2016 [140]	6/9	Healthy	45.0, 55.1 (10), 309, China	*ChREBP*	LC-MS	BP: indication of measurement.	PBC	No association.
Giannakopoulou et al. 2017 [141]	6/9	CABG surgery	75.8, 67 (59–73)^2^, 178, Greece	*CTH*	qMSP	HTN: SBP ≥ 140 mmHg and/or DBP ≥ 90 mmHg and/or anti-HTN usage.	WB	No association.
Guay et al. 2014 [142]	6/9	FH	100, N/A^2^, 30, Canada	*ADRB3*	Pyro	BP: measured seated after 3 minutes rest.	WB	Positive correlation between *ADRB3* methylation and SBP (r = 0.43, P < 0.05) and DBP (r = 0.45, P = 0.04).
Gu et al. 2016 [143]	6/9	HTN	39.6, 56.3 (8.2), 192, China	*CYP11B2*	Pyro	HTN: SBP ≥ 140 mmHg and/or DBP ≥ 90 mmHg and/or anti-HTN usage.	WB	No association.
Huang et al. 2016 [55]	7/9	JPS Family Follow-up	0, 32 (N/A)^2^, 256, Israel	*ABCA1, INS-IGF2, LEP, HSD11B2, NR3C1*	LC-MS	BP: indication of measurement.	PBC	Positive association between *NR3C1* methylation and DBP (β = 0.3, P = 0.01).
Jin et al. 2018 [144]	5/9	HTN	59.2, 48.8 (2.1), 76, China	*MFN2*	MALDI-TOF	HTN: SBP ≥ 130 mmHg and/or DBP ≥ 85 mmHg and/or anti-HTN usage.	WB	Lower C base methylation in HTN vs NT (P < 0.0001).
Jin et al. 2022 [145]	7/9	CHD	64.1, 61 (53.5–70)^2^, 553, China	*ACTB*	MALDI-TOF	BP: indication of measurement.	PBC	Positive correlation between *ACTB* methylation and HTN (ρ = 0.517 P = 0.046).
Koochakkhani et al. 2021 [59]	6/9	HTN	75, 43.5 (1.6), 20, Iran	*CBS, MTHFR*	Pyro	HTN: SBP ≥ 140 mmHg and/or DBP ≥ 90 mmHg and/or anti-HTN usage.	PBC	*CBS* methylation at 1 CpG was associated with HTN (OR = 5.3, 95%Cl: 0.895, 31.393, P = 0.047).
Krishnan et al. 2017 [146]	5/9	HTN	60, 42.6 (5.2), 20, India	*MIR-510*	MSP	HTN: SBP ≥ 140 mmHg and/or DBP ≥ 90 mmHg and/or anti-HTN usage.	WB	Higher *MIR510* methylation was higher in HTN vs NT controls (P < 0.05).
Kou et al. 2023 [147]	7/9	POUNDS Lost Trial	38.8, 50.7 (9.0), 672, USA	*LINC00319ATP2B1LMNA*	MCC-seq	BP: 3 measurements separated by 30 seconds after 5 min rest.	PBC	Per SD higher regional DNAm at *LINC00319*, was associated with 1.481 mmHg greater 24-month SBP reduction (P = 0.020) and 1.096 mmHg greater 24-month DBP reduction (P = 0.009) in low-fat diet group alone.
Li et al. 2020 [148]	8/9	Discovery:Gusu cohortValidation: CATIS	Discovery: 48.0, 55.7 (8.9), 2498, China Validation: 40.1, 63.5 (12.0), 1771 China	*NPPA*	BSeq	HTN: SBP ≥ 140 mmHg and/or DBP ≥ 90 mmHg and/or anti-HTN usage. BP: measured 3 times after 5 minutes rest.	PBC	Discovery: Average *NPPA* methylation was associated with HTN (P = 0.008) and SBP at 1 CpG site (P = 0.007). Validation: Average *NPPA* methylation was associated with SBP (P = 0.031), DBP (P = 0.002) and HTN at 5 CpG sites (P = 0.002).
Li et al. 2021 [149]	6/9	Hyperlipidemia	39.3, 51.3 (8.1), 211, China	*ABCA1*	BSeq	BP: measured 3 times at seated with 30 minutes rest.	PBC	*ABCA1* was associated with SBP and DBP (all P < 0.05). Positive correlation between *ABCA1_B* mean methylation and SBP (P < 0.05).
Li et al. 2022 [57]	8/9	HTN	45.3, 55.0 (9.1), 1034, China	*AGTR1*	qMSP	HTN: SBP ≥ 140 mmHg and/or DBP ≥ 90 mmHg and/or anti-HTN usage.	WB	*AGTR1* methylation was associated with HTN (OR = 0.946, 95%Cl: 0.896-0.999, P = 0.047).
Li et al. 2023 [150]	7/9	HTN	47.8, 58.9 (12.9), 343, China	*APP*	MassARRAY	BP: indication of measurement. HTN: SBP ≥ 140 mmHg and/or DBP ≥ 90 mmHg and/or anti-HTN usage.	PBC	Lower *APP* CpG_10 methylation in HTN vs NT (P = 0.043). 1% increase in CpG_10 methylation, HTN risk decreased by 32.4% (OR 0.676, 95%CI 0.467–0.977, P = 0.037). Higher *APP* CpG_19 methylation in HTN vs NT (P = 0.007). 1% increase in CpG_19 methylation, HTN risk increased by 4.1% (OR 1.041, 95%CI 1.002–1.081, P = 0.039). 1% increase in CpG_1 methylation, HTN risk decreased by 8% (OR 0.920, 95%CI 0.860–0.984, P = 0.015). Positive correlation between CpG_19 and SBP (r = 0.2, P = 0.03).
Lin et al. 2017 [58]	8/9	HTN	51.4, 40.8 (16.9), 326, China	*AGTR1*	MSP	HTN: SBP ≥ 140 mmHg and/or DBP ≥ 90 mmHg and/or anti-HTN usage.	Saliva	Lower *AGTR1* methylation within HTN vs NT (P < 0.001).
Li-Tempel et al. 2016 [53]	6/9	Healthy	100, 23.0 (2.8), 105, Germany	*GR*	Pyro	BP: indication of measurement.	WB	*GR* methylation was associated with SBP (P = 0.008) and DBP (P = 0.019).
Lopez-Legarrea et al. 2013 [151]	5/9	RESMENA	NA, NA, 46, Spain	*SERPINE1*	450 K	BP: measured to WHO criteria.	WBC	No association.
Macías-Gonzalez et al. 2018 [152]	7/9	Bariatric Surgery	RYGB: 31.3, 42.6 (8.7), 31, Spain LSG: 37.9, 46.9 (9.5), 29, Spain	*NF-kB1*	Pyro	BP: measured twice seated with an interval of 5 minutes between measurements.	PBMC	Higher NFKB1 methylation was associated with reduction in DBP in individuals who underwent RYGB (P = 0.012).
Mansouri et al. 2022 [153]	6/9	Chest pain	52.2, 54.8 (8.1), 110, Iran	*ABCA1*	MSP	BP: indication of measurement/medical history.	WB	No association.
Mao et al. 2016 [154]	8/9	HTN	35, 58.1 (7.9), 180, China	*SCNN1A*	Pyro	HTN: SBP ≥ 140 mmHg and/or DBP ≥ 90 mmHg and/or anti-HTN usage.	PBC	Higher *SCNN1A* methylation in HTN vs NT (P = 0.001).
Mao et al. 2017 [155]	8/9	HTN	39.5, 56.7 (8.7), 192, China	*TLR2*	Pyro	HTN: SBP ≥ 140 mmHg and/or DBP ≥ 90 mmHg and/or anti-HTN usage. BP: measure twice at reast 10 minutes apart.	PBC	Lower *TLR2* methylation at 1 CpG in HTN vs NT (P = 0.009). 1 CpG was associated with HTN (OR = 1.10, 95%Cl: 1.021, 1.161; P = 0.009) and was negatively correlated with SBP (r = -0.304; P < 0.001) and DBP (r = -0.329; P < 0.001).
Mao et al. 2017 [156]	7/9	HTN	39.5, 56.7 (8.7), 192, China	*IL-6*	Pyro	HTN: SBP ≥ 140 mmHg and/or DBP ≥ 90 mmHg and/or anti-HTN usage. BP: indication of measurement.	PBC	Higher *IL-6* methylation at 1 CpG in HTN vs NT (P = 0.004). Lower *IL-6* methylation at 1 CpG in HTN vs NT (P = 1.06E-07). 1 CpG was associated with HTN (OR = 1.11, 95%Cl: 1.036-1.193, P = 0.004). These CpGs were further negatively correlated with SBP and DBP (both P < 0.05).
Marotta et al. 2021 [157]	6/9	Moli-sani	49.2, 55.4 (11.7), 1160, Italy	*NMU76-F1, NMU76-F2, NMU32-F1, NMU32-F2*	Pyro	HTN: SBP ≥ 140 mmHg and/or DBP ≥ 90 mmHg and/or anti-HTN usage.	WBC	No association.
Meng et al. 2017 [158]	6/9	Healthy	84.6, 45.1 (7.4), 162, China	*NET*	Pyro	HTN: SBP ≥ 140 mmHg and/or DBP ≥ 90 mmHg and/or anti-HTN usage.	PBC	No association.
Milagro et al. 2011 [159]	5/9	Obesity	100, NA^2^, 25, Spain	*WT1*	MALDI-TOF	BP: measured 3 times with average taken.	PBC	Positive association between *WT1* methylation and intervention induced changes in DBP (all P < 0.05).
Milagro et al. 2012 [160]	5/9	Obesity	0, 39 (14), 60, Spain	*CLOCK*	MALDI-TOF	BP: measured to WHO criteria.	WBC	*CLOCK* methylation at 3 CpGs was associated with SBP (all P < 0.05). 1 CpG of *CLOCK* was associated with DBP (all P < 0.05).
Mo et al. 2020 [161]	5/9	MR: MEGASTROKE (MS) BSGS LBC UKB CC: CATIS SMSS	MR: NA^3^, NA^2^, 841348, EU CC: **IS Cohort**: 53.4, 61.0 (11.87), 2476, China **HTN sub-cohort:** 52.0, 66.1 (11.87), 1269, China	*CASZ1*	Next-Gen Seq	BP: Three consecutive BP measures taken after 30 minutes rest with average taken. HTN: SBP ≥ 140 mmHg and/or DBP ≥ 90 mmHg and/or anti-HTN usage.	MR: **MS:** NA^4^ **BSGS:** WB **LBC:** PBC **UKB:** WB CC: WBC	MR: discovery through epigenome-wide analysis CC (including IS and HTN sub-cohort): *CASZ1* at 2 CpG sites were significantly associated with SBP and DBP (P < 1.0E-03). Mean methylation of *CASZ1* was observed to be hypomethylated in HTN (P = 1.61E-03). Mean *CASZ1* methylation was also negatively correlated with SBP (r^2^ = 0.01, P < 0.0001) and DBP (r^2^ = 0.009, P < 0.0001).
Omar et al. 2019 [54]	5/9	Pre HTN	57.4, 31 (7)^2^, 160, Malaysia	*IL-6*	MethylLight qPCR	HTN: SBP ≥ 140 mmHg and/or DBP ≥ 90 mmHg and/or anti-HTN usage.	WB	Lower *IL-6* in Pre-HTN vs NT controls (P < 0.001). Inverse correlation between *IL-6* and all BP parameters (SBP = P < 0.05, DBP = P < 0.05, MAP = P < 0.05).
Osum et al. 2024 [162]	4/9	HTN	60.3, 60.0 (13.6) 78, Cyprus	*KLOTHO, ARNTL*	MSP	HTN: SBP ≥ 140 mmHg and/or DBP ≥ 90 mmHg and/or anti-HTN usage.	PBC	No association.
Peng et al. 2014 [163]	7/9	CHD	58, 61.3 (9.2), 139, China	*ABCG1, GALNT2, HMGCR*	MSP	BP: medical records.	PBC	No association.
Roberts et al. 2022 [164]	6/9	HTN	Discovery: 50.2, 44 (7), 281, USA Validation: 53.8, 42 (7), 117, USA	*ZC3H4*	BULLET-Seq	Clinical BP: measured 4 times after 2 minutes after 10-minute rest. 24 h-BP: BP was measured over a 24 hr period every 20 minutes (AM) or every 45 minutes (PM). HTN: SBP ≥ 140 mmHg and/or DBP ≥ 90 mmHg and/or anti-HTN usage.	WB	*ZC3H4* was associated with SBP 24 hr avg (α = 4.7, P = 0.0492), DBP 24 hr avg (α = 3.2, P = 0.0257) and MAP 24 hr avg (α = 3.5, P = 0.0354).
Shi et al. 2023 [165]	6/9	Discovery: GusuReplication: CATIS	Discovery: 38.5, 55.7 (8.9), 2498, China Replication: 54.0, 63.5 (12.0), 1771, China	*CORIN*	WGBS	BP: measured 3 times sitting after 5 minutes rest. HTN: SBP ≥ 140 mmHg and/or DBP ≥ 90 mmHg and/or anti-HTN usage	PBC	Discovery: Negative association between 9 CpG and SBP and DBP (q < 0.05). Hypermethylation of CpG1 (P = 0.018), CpG2 (P = 0.021) and CpG6 (P = 0.022) was associated with prevalent HTN risk before multiple correction (q > 0.05). Replication: Negative association between 9 CpGs and SBP (q < 0.05). Negative assocation between 8 CpGs and DBP (q < 0.05). 6 CpGs associated with lower risk of prevalent HTN risk (q < 0.05).
Turcot et al. 2013 [166]	6/9	Obesity	42.4, 35.4 (7.5), 105, Canada	*DPP4*	MSP	BP: measured sitting after 5 minutes rest. HTN: SBP ≥ 130 mmHg and/or DBP ≥ 85 mmHg.	VAT	No association.
Wang et al. 2014 [167]	5/9	T2DM	51.9, 52.9 (9.7), 54, China	*MIR-375*	MALDI-TOF	BP: indication of measurement.	WB	No association.
Wang et al. 2019 [60]	7/9	HTN, Stroke	58.8, 66.2 (9.8), 243, China	*CBS*	qMSP	HTN: SBP ≥ 140 mmHg and/or DBP ≥ 90 mmHg and/or anti-HTN usage.	PBC	Higher *CBS* methylation in HTN vs NT (P < 0.001). *CBS* methylation was associated with HTN (OR = 1.035, 95%Cl: 1.025, 1.045).
Wang et al. 2019 [168]	6/9	HTN	NA, NA, 243, China	*MTHFD1*	MSP	HTN: SBP ≥ 140 mmHg and/or DBP ≥ 90 mmHg and/or anti-HTN usage.	WBC	No association.
Wang et al. 2023 [169]	6/9	QTR	N/A, 52 (40, 66), 60, China	*COL5A1, WNT3A*	MALDI-TOF	HTN: SBP ≥ 140 mmHg and/or DBP ≥ 90 mmHg and/or anti-HTN usage.	WB	Positive association between 1 CpG of *COL5A1* and HTN (P = 0.048). Positive association between 3 CpGs of *WNT3A* and HTN (P = 0.003, P = 0.003 and P = 0.022).
Wei et al. 2016 [170]	6/9	IHD	NA^3^, NA^2^, 673, China	*IL-6*	Pyro	HTN: self-reported.	WBC	No association.
Xu et al. 2019 [171]	6/9	HTN	49.8, 66.2 (8.8), 241, China	*SHMT1*	qMSP	HTN: SBP ≥ 140 mmHg and/or DBP ≥ 90 mmHg and/or anti-HTN usage.	WB	Higher *SHMT1* methylation in HTN vs NT controls (P < 0.001).
Xu et al. 2019 [64]	7/9	HTN	50.6, 66.4 (9.3), 243, China	*MTHFD1*	qMSP	HTN: SBP ≥ 140 mmHg and/or DBP ≥ 90 mmHg and/or anti-HTN usage.	WB	Higher *MTHFD1* methylation in HTN vs NT controls (P < 0.001).
Xu et al. 2020 [29]	7/9	HTN	55.3, 66.4 (9.3), 691, China	*DHFR*	qMSP	HTN: SBP ≥ 140 mmHg and/or DBP ≥ 90 mmHg and/or anti-HTN usage.	WB	Higher *DHFR* methylation in HTN vs NT controls (P < 0.001).
Xu et al. 2020 [172]	6/9	HTN Stroke	48.12, 66.6 (9.36), 509, China	*MTHFR*	MSP	HTN: medical history	WB	Higher *MTHFR* methylation in HTN vs IS (P < 0.0001).
Xu et al. 2021 [173]	7/9	CAD	57.9, 60.4 (9.1), 1006, China	*ANRIL*	MSP	BP: medical history.	WB	Positive association between *ANRIL* and SBP (P = 0.013) and DBP (P = 0.028).
Xu et al. 2023 [174]	6/9	CSVD	55.4, 70.0 (13.0), 101, China	*PER, CRY1*	MSP	BP: BP was measured every 30 min during the day and every hr at night.	WB	No association.
Yang et al. 2016 [175]	5/9	T2DM	43.5, 60.1 (8.41), 85, China	*MTHFR*	MSP	BP: indication of measurement.	WB	No association.
Zhang et al. 2013 [62]	7/9	HTN	45.9, 50.2 (5.3), 61, China	*ADD1*	Pyro	HTN: SBP ≥ 140 mmHg and/or DBP ≥ 90 mmHg and/or anti-HTN usage.	PBC	Lower *ADD1* methylation in HTN vs NT (P = 0.026).
Zhang et al. 2013 [176]	6/9	Gen Pop	41.1, 43.6 (n/a)^2^, 517, USA	*FABP3*	MALDI-TOF	BP: indication of measurement.	PBC	*FABP3* methylation was associated with DBP (P = 0.00129).
Zhang et al. 2022 [177]	7/9	SMSS	52.2, 66.5 (11.5), 1241, China and Europe	*PRDM6, IGFBP3, SYT7, PDE3A, TBX2, C17orf82, HDAC9*	MethylTarget™	BP: measured 3 times with 30 s intervals in-between after sitting at rest for 30 minutes. HTN: SBP ≥ 140 mmHg and/or DBP ≥ 90 mmHg and/or anti-HTN usage.	WB	BP: 20 CpG sites were associated with SBP and 72 CpG sites were associated with DBP (FDR < D0.05). HTN: 13 CpG sites were associated with HTN (FDR < 0.05).
Zhong. 2016 [178]	8/9	HTN	34, 56.3 (7.7), 282, China	*SCNN1B*	Pyro	HTN: SBP ≥ 140 mmHg and/or DBP ≥ 90 mmHg and/or anti-HTN usage.	WBC	Higher *SCNN1B* methylation at 1 CpG IN HTN vs NT controls (P = 0.030). 1 CpG was associated with incident HTN (OR = 1.185, P = 0.015). Lower methylation at 1 CpG HTN vs NT (P = 0.008). This CpG was associated with incident HTN (OR = 0.663, P = 0.001) and prevalent HTN (OR = 0.589, P = 1.66E-04).

*BC* buffy coat, *BP* Blood pressure, *BSeq* bisulfite sequencing, *BULLET-Seq* bisulfite/bisulfite-specific polymerase chain reaction ultraplex targeted sequencing, *CABG* coronary artery bypass graft, *CAD* coronary artery disease, *CATIS* China antihypertensive trial in acute ischemic stroke, *CHD* coronary heart disease, *CSVD* Cerebral small vessel disease, *CVD* cardiovascular disease, *DBP* Diastolic blood pressure, *Gen pop* general population, *FH* familial hypercholesterolemia, *HBP* High blood pressure, *HRM* high resolution melt curve analysis, *HTN* hypertension, *IHD* Ischemic heart disease, *KAROLA* Langzeiterfolge der Kardiologischen Anschlussheilbehandlung, *LBC* Lothian birth cohort, *LC-MS* liquid chromatography mass spectroscopy, *LSG* Laparoscopic sleeve gastrectomy, *MALDI-TOF* matrix-assisted laser desorption/ionization time of flight, *MAP* Mean arterial pressure, *MDD* major depressive disorder, *MEGASTROKE* multi-ancestry genome-wide association study of 520,000 subjects identifies 32 loci associated with stroke and stroke subtypes, *MCC-seq* high-resolution methyl-capture sequencing, *MetS* metabolic syndrome, *MS* mass spectrometry, *MSP* methylation specific polymerase chain reaction; MS RT-PCR, methylation specific reverse-transcription polymerase chain reaction, *NAS* normative aging study, *NT* normotensive, *PBC* peripheral blood cells, *PBMC* peripheral blood mononucleocytes, *PP* Pulse pressure, *Pyro* pyrosequencing, *qMSP* quantitative methylation specific polymerase chain reaction, *OSA* obstructive sleep apnoea, *RESMENA* reduction of metabolic syndrome in Navarra, *RYGB* Roux-en-Y gastric bypass, *SBP* Systolic blood pressure, *SPHERE* Susceptibility to Particle Health Effects, miRNAs and Exosomes, *SAT* subcutaneous adipose tissue, *SMSS* Suzhou Metabolic Syndrome Study, *T2DM* type 2 diabetes mellitus, *TWB* Taiwan Biobank, *UKB* UK biobank, *VAT* visceral adipose tissue, *WB* whole blood, *WBC* white blood cells, *WGBS* Whole Genome Bisulfite Sequencing

^1^Quality assessment determined using Newcastle-Ottawa scale. Highest score 9/9

^2^Age is either given as median (IQR), SD is unavailable, or age is not described

^3^Percentage male not described

^4^Tissue not specified

**Table 3:** Associations between blood pressure or hypertension and epigenome-wide DNA methylation

Author, Year	Quality^1^	Population	Male (%), Age, sample size, country	Platform	Tissue type	Number of CpGs/DMRs detected	Top gene(s) annotated	Definition of HTN/BP	Associations between epigenome-wide DNA methylation and HTN and/or BP.
Bai et al. 2022 [122]	8/9	HTN/Pre-HTN	Discovery: 1:1 ratio^3^, 58.2 (5.1), 132, China Validation: 1:0.8 ratio^3^, 59.96 (5.5), 203, China	450 K	PBC	Discovery: 25 CpGs	*MBLP, PVT1, CCDC85C, COLEC11, MAML2, PYROXD1, OVGP1, ADAMTS19, MCF2L, PSORS1C1*	HTN: Double-arm SBP and DBP values ≥ 160 mmHg and ≥110 mmHg respectively, or SBP ≥ 140 mmHg and/or DBP ≥ 90 mmHg after receiving anti-HTN treatment.	Discovery: 25 CpG sites with a selection criterion of a DiffScore >13 and ∆β > 0.1 identified though overlap of differentially methylated CpGs between HTN vs healthy controls and between patients with prehypertension who progressed to hypertension vs those who did not progress to hypertension. Validation: by pyrosequencing.
Boström et al. 2016 [179]	6/9	Discovery: Bariatric surgery Validation: ArrayExpress databases^d^	Discovery: 63.6, 46.9 (11.9), 11, Switzerland Validation: 50.9, 64.9 (13.2), 540, N/A^4^	450 K	WB	Discovery: 22 CpGs Validation:2 CpGs	*MCC1, SKOR2, VGLL3, WSB1, EHMT2, TCF12, MAP2K4, FAM54A, SORBS1, DDX55, ATXN1L, EFEMP1, CRKL, STEAP3, CPEB4, RHOBTB2, ATXN1L, TSSK1B, SPPL2A, BHLHA9, CHCHD5, NRTN*	BP: Measured once after overnight fast, in sitting position, after 5 min relaxation, before and six months after surgery. HTN: Yes/No to HTN provided by original cohort study author.	Discovery: Methylation changes at 22 CpGs correlated with SBP six months after RYGB surgery. Validation: 2 CpG sites (*SKO2*, P = 2.79E-02; *EHMT2*, P = 2.30E-02) significantly hypomethylated in HTN.
Caspers et al. 2019 [180]	5/9	LLSLFH	100, 49.0 (11.8), 68, Belgium	450 K	WB	5 CpGs	*MECOM, CACNB2, CETP, G6PC2, SH2B3*	BP: BP was measured three times in the morning seated. Average (ABP) of SBP and DBP was used.	5 CpGs associated with ABP or ΔABP promoter region overlapping with genetic variants linked to 1 or more MetS phenotypes. No associations with ABP or ΔABP at FDR P < 0.05.
Chen et al. 2023 [130]	7/9	TWB	47.9, 49.7 (0.43), 1442, Taiwan	850 K	WB	1 CpG	*GPNMB*	BP: indication of measurement.	HTN patients had ↑ cg17274742 methylation.
Ciantar et al. 2024 [181]	6/9	Discovery: YFS Replication: LURIC	Discovery: 50.6, 43.0 (37.0, 46.0), 969, Finland Replication: 69, 62.8 (10.7), 2371, Germany	850k	PBC	Discovery: 1 DMR Replication: 1 CpG	Discovery: *LDHAL6A* Replication: *LINC01237*	BP: 3 successive measurements taken on right arm of the participants after 5-min rest while seated.	Discovery: 1 DMR associated with SBP (P = 3.8E-02). Replication: 1 CpG associated with DBP (P < 1.2E-02).
Cronje et al. 2020 [182]	7/9	PURE-SA-NW	100, 63 (N/A)^2^, 120, Botswana	850k	WB	1 CpG	*MED13L*	BP: SBP and DBP measured twice in seated or upright position with 5-minute interval.	2% methylation ↑ in cg03621504 was associated with a 10 mmHg increase in SBP.
da Silva Rodrigues et al. 2024 [183]	7/9	Older women	0, 60.7 (4.1), 40, Brazil	850k	PBC	35 CpGs	Top CpGs: *ACE, ABCA7, SORL1, IQCK*,	BP: indication of measurement.	35 CpGs were differentially methylated in response to reduced SBP and DBP (P < 0.05).
Das et al. 2016 [184]	7/9	Discovery: GOLDN Validation: BHS	Discovery: 51.5, 53.5^2^, 846, USA Validation: **BHS EU:** N/A^3^, 44.1 (4.1), 603, USA **BHS AfriA:** N/A^3^, 43.5 (4.5), 269, USA	450k	Discovery: CD4 + T cells Validation: WB	Discovery: 2 CpGs Validation: **BHS EU:** 2 CpGs **BHS AfriA:** 2 CpGs	*CPT1A*	BP: BP measured seated after 5 minutes rest.	Discovery: ↓ methylation at cg00574958 associated with meeting MetS BP criteria (HTN) (P < 0.0001). ↓ methylation cg17058475 associated with HTN (P < 0.05). Validation: Both CpGs significantly associated with MetS in both BHS EU and BHS AfriA.
Fernández-Sanlés et al. 2018 [27]	7/9	Discovery: REGICOR Validation: FHS-OS	Discovery: 48.2, 58.5 (9.3), 465, Spain Validation: 46.9, 63.5 (7.3), 1823, USA	450k	Discovery: PBC Validation: BC	Discovery: N/A Validation: N/A	N/A	BP: SBP and DBP measured twice with lower value recorded.	Discovery, Validation and Meta-analysis: No association with SBP or DBP in either cohort or in the overall meta-analysis at P < 7.81E-04.
Hong et al. 2023 [28]	7/9	CNTR	Cross sectional: 68.7, 49.9 (12.2), 1072, China Longitudinal **Baseline:** 60.7, 50.2 (10.2), 308, China **Follow up:** 60.7, 54.9 (10.2), 308, China	450 K 850 K	PBC	Cross sectional: 33 CpGs Longitudinal: 9 CpGs	Cross sectional: *GALE, SLC1A5, STAB1, EGFL7, ANKRD34B, LOC100132354, CPT1A, LMNA, SCNN1A, CDC42BPB, DNMT3B, ADAM8, LRRC33, VPS37B, TSPAN2, MKLN1, TXNIP, CDK6, SLC7A11, SLC43A1, SLC9A3R1, RAB7A, FAM117A, B3GALT4, FAM60A, PHGDH, ZDHHC18* Longitudinal: *CPT1A, ANKRD34B, DNMT3B, MKLN1, SLC4341, LMNA*	BP: BP measured twice on right arm in sitting position after 5 min of rest with the average calculated. If measurements differed by >10 mmHg another measurement was taken. HTN: SBP ≥ 140 mmHg and/or DBP ≥ 90 mmHg and/or antihypertensive medication usage.	Cross sectional: 7 previously reported CpGs significantly associated with SBP and 3 CpGs significantly associated with DBP in total cohort. Discordant MZ twin analysis outlined 11 CpGs associated with SBP and 18 CpGs associated with DBP. Longitudinal: 2 CpGs significant comparing baseline DNA methylation to follow-up SBP. 3 CpGs significant comparing baseline SBP to follow-up DNA methylation. 3 CpGs significant comparing baseline DNA methylation to follow-up DBP. 1 CpG significant comparing baseline DBP to follow-up DNA methylation.
Huan et al. 2019 [185]	7/9	FHS-OS FHS-TG	FHS-OS: 46, 66.4 (8.9), 2648, USA FHS-TG: 48, 45.4 (7.8), 1522, USA	450 K	BC	14 CpGs	*CNNM2, SIPA1, FAM109A, LASS5, SOX7, OR4B1, HIVEP3, CLN8, SDCCAG8, PHACTR1, PRSS42, TSHZ3, MACROD1, HSPB7, TRAPPC9, SPINK8, SEMA7A, PLAC9, CLN8, IRF1, KCNH2, SCAMP2, FLII, NME6*	BP: BP measured twice by a physician; average taken.	Mendelian randomisation analysis identified 37 causal CpGs for BP. 29 CpGs associated with SBP and 22 CpGs associated with DBP.
Kato et al. 2015 [186]	8/9	Discovery: LOLIPOP IA Validation: LOLIPOP IA 2 LOLIPOP EU LifeLines DEEP RS-BIOS KORA	Discovery: **LOLIPOP1A:** 74.2, 51.9 (10.2), 1904, UK (S.Asian) Validation: **LOLIPOP1A 2:** 47.0, 51.8 (10.2), 1373, UK (S. Asian) **LOLIPOPEU:** 50.6, 55.6 (9.5), 166, UK **LifelinesDeep:** 42.3, 45.5 (13.3), 752, Netherlands **RS-BIOS**: 42.5, 67.7 (5.9), 762, Netherlands **KORA**: 48.9, 61.0 (8.9), 1727, Germany	450 K	**LOLIPOP:** WB **LifelinesDeep:** WB **RS-BIOS:** WB **KORA:** WB	Discovery: 28 CpGs	*CASZ1, MTHFR, OSR1, KCNK3, ULK4, PRDM8, ARHGAP24, NPR3, PRDM6, ABLIM3, EBF1, TTBK1, PLEKHG1, IGFBP3, TBC1D12, TRIM8, ADRB1, SYT7, ARHGAP42, ADAMTS8, GALNT4, FAM109A, RPP25, FES, DCAKD, HOXB3, TBX2, AMH*	HTN: SBP ≥ 160 mmHg and/or DBP ≥ 100 mmHg and/or antihypertensive usage. BP: measured to standardised protocols.	Discovery: 28/35 sentinel blood pressure SNPs associated with one or more CpGs at P < 3.8E-06. 10 CpGs associated with MAP, 6 CpGs associated with PP, 9 CpGs associated with SBP and 3 CpGs associated with DBP. Validation: All 28 CpGs replicated in further testing with consistent direction of effect.
Kazmi et al. 2020 [187]	7/9	SABRE	Trans-ancestry: 100, 51 (7.1), 712, UK EU: 100, 52 (7.2), 364, UK S.Asian: 100, 51 (7.0), 348, UK	450 K	PBC	Trans-ancestry: 9 CpGs 620 DMRs EU: 3 CpGs 435 DMRs S.Asian: 1 CpGs 247 DMRs	Trans-ancestry CpGs: *FHL2, MYO5C, ELOVL2, KLF14, AHRR, MYO1G, OR8U8;OR5AP2, PPP1R2, LOC100132354* Top 5 DMRs SBP trans-ancestry: *CUX1, LOC729156, ARHGAP12, C21orf129, NCRNA00112* Top 5 DMRs DBP trans-ancestry: *DDX10, UNKL, CD44, GNG7, DRNA5*, EU CpGs: *CEBPD, AHRR, C12orf44, AFAP1-AS1*, Top 5 DMRs SBP EU: *CUX1, LOC729156, ARHGAP12, C21orf129, SR140, CAMTA1, NDE1, ATP10D, IRF8, FJ39582* Top DMRs SBP EU: *BBS1, BCORL1, NCKAP5, DYNC1I1, MYLK, ACSL4, BANP, PRDM2, DNAJC8, SLC4A11* S.Asian *GALR2*, Top DMRs SBP S.Asian: *C17orf53, MTCH1, CRTAM, ELL2, BCOR, C3orf21, FAM194A, MSRA, TRPM1, ALDH15A1* Top DMRs DBP S.Asian: *ELL2, ALDH16A1, PID1, DDX10, CD44, ZNF77, ADAMTS5, MSRB3, FXN, SNORD113-7*	HTN: SBP ≥ 140 mmHg and/or DBP ≥ 90 mmHg and/or antihypertensive medication usage. BP: BP measured twice in sitting position after participant had urinated and rested for 5 minutes.	Trans-ancestry: 4 CpGs associated with SBP (P < 1.24E-07). After adjustment for cofounders no longer significant. 2 CpGs associated with DBP (P < 1.24E-07). After adjustment 1 additional CpG was identified. 2 CpGs associated with HTN (P < 1.24E-07). After adjustment these associations no longer remains significant. 395 DMRs (mapped to 326 genes) were associated with SBP. 237 DMRs (mapped to 157 genes) were associated with DBP FDR (P < 0.05). EU: 3 CpGs were associated with DBP (P < 1.24E-07). When adjusted 2 of these CpGs remained significant with consistent direction of effect. 1 additional CpG was identified. 348 DMRs (mapped to 291 genes) associated with SBP. 95 DMRs (mapped to 74 genes) associated with DBP FDR (P < 0.05). S.Asian: 1 CpGs associated with SBP (P < 1.24E-07). After adjustment not significant. 96 DMRs (mapped to 66 genes) associated with SBP. 186 DMRs (mapped to 135 genes) were associated with DBP FDR (P < 0.05).
Kho et al. 2020 [92]	6/9	GENOA	28.6, 63.6 (9.2), 1218, USA	450 K 850 K	PBC	3 CpGs	*LOC100132354, CPT1A, TXNIP*	BP: BP was measured twice in sitting position for 5 minutes, average taken. HTN: SBP ≥ 140 mmHg and/or DBP ≥ 90 and/or antihypertensive medication usage.	3 previously reported CpGs associated with BP (P < 0.0038). 2 CpGs associated with SBP while 1 CpG associated with DBP (P = 0.0006).
Kidambi et al. 2021 [188]	6/9	Post-menopausal women and men with HTN	N/A^3^, 51 (9), 50, USA	RBBS	T-Cells, Frozen arterioles	T Cells: 1 DMR Arterioles: 3 DMRs	T Cells: Chr12:34499685-34499731 Arterioles: *MESTIT1, MEST, NXPH4*, chr17:26578249-26578315	BP: BP measured three times 1 minute apart with the subject seated with after 5 minutes rest. HTN: SBP ≥ 130 mmHg and/or DBP ≥ 80 mmHg and/or antihypertensive medication usage.	T Cells: Changes in DBP (due to sodium restriction) were associated with one intergenic DMR (P_adj_=0.01). Arterioles: Changes in SBP associated with 1 DMR (*MESTIT1:MEST*, P_adj_ = 0.0067). Changes in DBP associated with 1 intergenic DMR (P_adj_=0.044). One DMR associated with change in DBP however did not remain significant after adjustment (P_adj_=0.075).
Kucher et al. 2017 [189]	4/9	CAH and AHTN	100, 55.5 (6.5), 6, Russia	27 K	WBC, CAAP	WBC: 3 CpGs CAAP: 3 CpGs	WBC: *MIR615, MIR7-3* CAAP: *MIR10B, MIR675*	HTN: Medical history	WBC: Duration of HTN associated with 3 CpGs (P < 0.05). CAAP: Duration of HTN associated with 3 CpGs (P < 0.05).
Lee et al. 2024 [190]	7/9	KARE HEXA	KARE: 52.9, 58 (47, 78), 1,526, S. Korea HEXA: 75.5, 52 (40, 60), 808, S. Korea	850 K	WB	KARE: 2 CpGs HEXA: 2 CpGs Overall Meta-analysis: 4 DMRs for both SBP and DBP	KARE: *CPT1A, SLC13A5* HEXA: *CPT1A, SLC13A5* Overall Meta-analysis: *SLC25A29, SLC16A11, ZNF835, CAST, CPT1A, ACSF3*	BP: indication of measurement HTN: SBP ≥ 130 mmHg and/or DBP ≥ 80 mmHg and/or antihypertensive medication usage	KARE: 2 CpGs associated with SBP (P < 4.41E-06). HEXA: 2 CpGs associated with SBP (P < 2.96E-03). Overall Meta-analysis: 4 DMRs associated with SBP (P < 1.49E-07). 4 DMRs associated with DBP (P < 2.49E-09).
Lim et al. 2017 [191]	7/9	Elderly Gen Pop	Discovery: 6.0, 73.3 (4.1), 50, South Korea Validation: 9.6, 72.3 (3.8), 52, South Korea	450 K	WB	2 CpGs	*ZKSCAN4, ZNF227*	BP: measured twice in sedentary position after 10 minutes rest with a 10 minute interval between measurements and average taken. HTN: self-reported medication information.	Discovery and validation combined: 10% ↑ in mean methylation of 2 CpGs was associated with a 4.0 mmHg increase in DBP. 10% ↑ in methylation of cg21194911 was associated with a 2.7 mmHg increase in DBP. 10% ↑ in methylation of cg21761427 was associated with 5.0 mmHg increase in DBP.
Lin et al. 2016 [192]	6/9	LBC1921 LBC1936	LBC1921: 40, 79.1 (0.6), 446, Scotland LBC1936: 51, 69.5 (0.8), 920, Scotland	450 K	WB	1 CpGs	*CLCN6*	BP: indication of measurement	*CLCN6* was correlated with SBP (r = -0.022) and DBP (r = 0.013).
Lu et al. 2020 [193]	7/9	LifelinesDEEP	42, 46 (36-55)^2^, 622, Netherlands	450 K	WB	4 CpGs	*MIR1249, SLC6A19, IL12RB2*	BP: 10 measurements over 10 minutes.	1 CpG associated with SBP and 3 CpGs associated with DBP (FDR < 5%).
Mens et al. 2020 [194]	7/9	RS-II-3 RS-III-2 RS-III-1	RS-II-3 + RS-III-2: 42.4, 67.5 (5.9), 717, Netherlands RS-III-1: 45.8, 59.8 (8.16), 721, Netherlands	450 K	PBC	109 CpGs	*MIR196A23B, MIR6773, MIR3941, MIR125B5P, MIR19085P, MIR4729, MIR6766, MIR489, MIR653, MIR4721*	BP: measured twice while sitting separated by count of pulse rate with average taken.	50 CpGs associated with SBP. 59 CpGs were associated with DBP in miRNAs identified via GWAS (P < 0.05).
Mo et al. 2020 [161]	8/9	MQTL: BSGS LBC CC: CATIS SMSS	MR: **BSGS:** 614 **LBC**: 1,366 IS Cohort: 53.4, 61.0 (11.87), 2476, China HTN sub-cohort: 52.0, 66.1 (11.87), 1269, China	450 K	MQTL: **BSGS:** WB **LBC:** PBC CC: WBC	510 CpGs	SBP: *CNNM2, NT5C2, SLC5A11, FES, TNRC6A, CASZ1, TNXB, FAM109A, ADAMTS8, HOXA13* DBP: *FAM109A, NOV, CPEB4, ZSCAN12L1, ZN389, CNNM2, ZSCAN16, SH2B3, CYP21A2, FES*	BP: Three consecutive BP measures taken after 30 minutes rest with average taken. HTN: SBP ≥ 140 mmHg and/or DBP ≥ 90 mmHg and/or antihypertensive usage	MR: 173 CpGs in 90 genes associated with SBP. 337 CpGs in 142 genes associated with DBP (P_SMR_ < 5.67E-07). IS cohort and HTN sub-cohort: Validated through Next-Gen Seq.
Nuotio et al. 2020 [93]	7/9	Discovery: DILGOM (FINRISK) Validation: NFBC1966	Discovery: **DILGOM:** 46.2, 51.9 (13.8), 517, Finland Validation: **NFBC1966:** 45.1, 31.0 (0.3), 670, Finland	450 K	Discovery: WBC Validation: WBC	16 CpGs	Discovery: *NUMB, GPS1, MYH14, FNDC3B, DOK2, GLIS3, FNDC2B, NBPF8, NBPF12, TXNIP, KCNK17* Validation: *ABCG1*	Discovery: BP: measured 3 times after seated at rest for 5 minutes with average of 2^nd^ and 3^rd^ measurement taken. Validation: BP: measured after 15-minute rest period.	Discovery: 7 CpGs associated with SBP. 8 CpG sites associated with DBP (P < 7.33E-06). Validation: 1 CpG associated with DBP which was not observed in the discovery analysis.
Pan et al. 2024 [195]	6/9	HTN	50.2, 44 (7), 281, USA	RBBS	WB	24 h-SBP: 108 DMR 24 hr DBP: 119 DMR 24 h-MAP: 104 DMR	Top DMR 24 h-SBP: *MMP23B:MMP23A, FAAP20, LDLRAD2:HSPG2* Top DMR 24 h-DBP: *CASZ1, MMP23B:MMP23A, PLXNA2* Top DMR 24 h-MAP: *MMP23B:MMP23A, PLXNA2*	Clinical BP: measured 4 times every 2 minutes after 10 minute rest. 24 h-BP: BP was measured over a 24 hr period every 20 minutes (AM) or every 45 minutes (PM).	108 DMR associated with 24 h-SBP, 119 DMR associated with 24 h-DBP and 104 associated with 24 h-MAP (FDR < 0.05).
Portilla-Fernandez et al. 2019 [196]	7/9	RS	24, 60.6 (5.3), 1450, Netherlands	450 K	PBC	3 CpGs	*ATG4B, ULK1*	BP: measured twice while sitting separated by count of pulse rate with average taken.	3 CpGs associated with SBP and 1 CpG associated with DBP (P < 5.63E-05).
Ramos-Lopez et al. 2018 [197]	6/9	MENA	36.1, 47.2 (14.1), 474, Spain	450 K	WBC	4 CpGs	*PPKAG2, BHLHE40, PER3, RORA*	BP: indication of measurement.	Negative correlation between 4 CpG sites and MAP (P < 0.05).
Richard et al. 2017 [84]	8/9	Discovery: ARIC, CHS, FHS, GENOA, GOLDN, LBC1936, NAS, RS-III, TwinsUK Validation: Amish, ARIC MESA RS-III SYS adults WHI-BAA23 WHI-EPMC	Discovery: **ARIC**: NA^3^, 56.6 (5.9), 2743, USA **CHS (AA)**: NA^3^, 73.0 (5.4), 196, USA **CHS (EA)**: NA^3^, 76.0 (5.1), 189, USA **FHS**: NA^3^, 66.4 (8.9), 2645, USA **GENOA**: NA^3^, 60.1 (8.4), 239, USA **GOLDN**: NA^3^, 48.8 (15.9), 822, USA **LBC1936**: NA^3^, 69.5 (0.8), 903, Scotland **NAS:** NA^3^, 72.5 (6.8), 674, USA **RS-III**: NA^3^, 59.7 (8.2), 727, Netherlands **TwinsUK:** NA^3^, 58.4 (9.3), 690, UK Validation: **AMISH**: NA^3^, 46.3 (13.6), 192, USA **MESA (AA):** NA^3^, 60.6 (9.2), 236, USA **MESA (EA):** NA^3^, 60.8 (9.6), 566, USA **MESA (HL):** NA^3^, 59.0 (9.5), 381, USA **RS-III:** NA^3^, 67.5 (6.0), 711, Netherlands **SYS adults:** NA^3^, 47.2 (4.9), 111, Canada **WHI-BAA23 (AA):** NA^3^, 62.8 (6.7), 666, USA **WHI-BAA23 (EA):** NA^3^, 68.4 (6.2), 965, USA **WHI-BAA23 (HL):** NA^3^, 62.3 (6.8), 333, USA **WHI-EMPC (AA):** NA^3^, 62.8 (7.0), 556, USA **WHI-EMPC (EA):** NA^3^, 64.7 (7.1), 1092, USA **WHI-EMPC (HL):** NA^3^, 61.6 (6.2), 315, USA	450 K	All (excluding GOLDN): WB GOLDN: CD4 + T Cells	Discovery: 31 CpGs Validation: 13 CpGs Overall Meta-analysis (Discovery + Validation): 126 CpGs	Discovery: *SKI, PHGDH, SLC7A11, TXNIP, SLC1A5, CNP, GALE, ZMIZ1, LOC100132354, CPT1A* Validation: *TSPAN2, PHGDH, TXNIP, SLC7A11, LOC132354, ZMIZ1, CPT1A, SLC1A5* Overall Meta-analysis (Discovery + Validation): *PHGDH, SLC7A11, TXNIP, SLC1A5, LOC100132354, SLC43A1, SARS, GARS, CPT1A, CSAD*	BP: measured in a sitting position after a period of rest and an average of sequential readings was taken.	Discovery: 31 CpG sites associated with both SBP and DBP (P < 1.0E-07). Validation: 13 of the 31 identified CpG sites associated with SBP and DBP (P < 0.0016). Overall Meta-analysis (Discovery + Validation): 126 CpG sites associated with SBP and DBP (P < 1.0E-07).
Roberts et al. 2022 [164]	6/9	HTN	Discovery: 50.2, 44 (7), 281, USA Validation: 53.8, 42 (7), 117, USA	RBBS	WB	24 h-SBP: 72 DMRs SBP day: 38 DMRs SBP night: 20 DMRs 24 h-DBP: 12 DMRs DBP day: 9 DMRs DBP night: 4 DMRs 24 h-PP: 22 DMRs PP day: 28 DMRs PP night: 2 DMRs 24 h-MAP: 34 DMRs MAP day: 7 DMRs MAP night: 5 DMRs	Top DMRs 24 hr SBP: *PRDM16, COLGALT1, ZC3H4, CFAP100, CASZ1* Top DMRs SBP day: *PRDM16, CASZ1, CASKIN1, COLGALT1, ZC3H4* Top DMRs SBP night: *OLFM2, ZC3H4, BAIAP3, CASZ1, FAM181B* Top DMRs 24 hr DBP: *ZC3H4, COLGALT1, CASZ1, FGF19, PRDM16* DBP day: *CASZ1, ZC3H4, COLGALT1, PRDM16, FGF19* Top DMRs DBP night: *ZC3H4, FGF19, CASZ1* Top DMRs 24 h PP: *ALX4, PRR35, CASKIN1, CFAP100, PMPCA* Top DMRs PP day: *CASKIN1, PMPCA, PRDM16, ALX4, CFAP100*, Top DMRs PP night: *ALX4* Top DMRs 24 h MAP: *PRDMB1491, B152516, PRDM16, COLGALT1, ZC3H4* Top DMRs MAP day: *PRDM16, CASZ1, COLGALT1, CFAP100, MPRIP* Top DMRs MAP night: *ZC3H4, CASZ1, FGF19*	Clinical BP: measured 4 times every 2 minutes after 10 minute rest. 24 h-BP: BP was measured over a 24 hr period every 20 minutes (AM) or every 45 minutes (PM). HTN: SBP ≥ 140 mmHg and/or DBP ≥ 90 mmHg and/or antihypertensive medication usage.	Discovery: 72 DMRs associated with 24 hr SBP (FDR < 0.05). 38 DMRs associated with SBP day (FDR < 0.05). 20 DMRs were associated with SBP night (FDR < 0.05). 12 DMRs associated with 24 hr DBP (FDR < 0.05). 9 DMRs associated with DBP day (FDR < 0.05). 4 DMRs associated with DBP night (FDR < 0.05). 22 DMRs associated with 24 hr PP (FDR < 0.05). 28 DMR associated with PP day (FDR < 0.05). 2 DMRs associated with PP night (FDR < 0.05). 34 DMRs associated with 24 MAP (FDR < 0.05). 7 DMRs associated with MAP day (FDR < 0.05). 5 DMRs associated with MAP night (FDR < 0.05). Validation: by BULLET-Seq
Si et al. 2021 [198]	8/9	CKB	56.4, 49.5^2^, 982, China	850k	PBC	2 CpGs	*SNX30, IMPDH2, PEMT*	BP: measured twice using standardised protocols.	Participants in Q1 of methylation had lower SBP and DBP when compared to those in Q4 (P < 0.05)
Sprague et al. 2022 [199]	6/9	Gen pop	50.0, 67.1 (60.1-72.6)^2^, 112, Croatia	850k	WB	22 CpGs	*C16orf72, BTN3A2, ACIN1, CUL2, MIEF1, TSTD2, NCBP1, CANT1, KRTCAP2, TRIM46*	BP: measured 3 times with the second and third measurements averaged.	22 CpGs associated with SBP(q < 0.01).
Wang et al. 2023 [169]	6/9	QTR	N/A^3^, 52 (40, 66), 60, China	RBBS	WB	SBP: 31 CpGs, 8 DMRs DBP: 43 CpGs, 12 DMRs	Top SBP CpGs: *SRRM1P2, MIR3147* Top SBP DMRs: *NFATC1, CADM2, LRAT, TUBA3C, LOC100507377* Top DBP CpGs: *WNT3A, PLCH2, SIM1* Top DBP DMRs: WNT3A, NKX2-4, TMEM114, ZBTB25, CNOT10	BP: measured 3 times with 1 minute interval and averaged.	31 CpGs and 8 DMRs associated with SBP (P < 1E-04). 43 CpGs and 12 DMRs associated with DBP (P < 1E-04).
Wu et al. 2022 [200]	6/9	TWB	46.6, 49.0 (40.0–58.0)^2^, 1636, Taiwan	850k	WB	1 CpG	*KLF14*	BP: indication of measurement. HTN: SBP ≥ 140 mmHg and/or DBP ≥ 90 mmHg and/or self-reported history of HTN.	1 CpG associated with HTN (P = 0.0041), SBP (P = 0.0126), and Mean BP (P = 0.0212).
Xiao et al. 2022 [71]	8/9	Gen pop	42.9, 78.5 (16.1), 280, China	850k	WBC	11 DMRs	*FMOD, TTC22, CEPZOS, HOPX, CCDC125, MIR886, HLA-DRB1, HOXA5, LINC00857, GLIPR1L2*	BP: measured 2-3 times after sat at rest for 5 minutes. HTN: SBP ≥ 140 mmHg and/or DBP ≥ 90 mmHg and/or antihypertensive medication usage.	11 DMRs associated with HTN (FDR < 0.05).
Yao et al. 2024 [201]	6/9	QTR	53, 52 (40, 66), 60, China	RBBS	WB	SBP: 9 CpGs DBP: 33 CpGs	*C10orf71-AS1, NDUFB5P1, KRT80, BAI2, ABCA2, PEX11G, FGF4, LMNB2, KDM4B, DNASE1/TRAP1, TFCP2L1, SLC25A19, HMCN2, RSPH6A, PTPRN2, PNPLA7, KRI1, ADORA2B, NAA60, STIL, CTNNBIP1, LINC01070, KIF26A, XKR6*	BP: measured using standard protocols	9 CpGs associated with SBP (P < 0.05). 33 CpGs associated with DBP (P < 0.05).
Zhang et al. 2022 [176]	8/9	HRS	41.5, 70.5 (9.5), 3070, USA	850k	WB	9 CpGs	*PHGDH, SLC7A11, LOC100132354, ZMIZ1, SLC1A5*	BP: measured 3 times with 45 s intervals between. HTN: antihypertensive medication usage.	In a fully adjusted model 5 CpGs (out of 13 previously reported CpG sites) were associated with SBP (P < 0.0038). 7 CpGs associated with DBP (P < 0.0038).

*ARIC* Atherosclerosis Risk in Communities, *Amish* Amish Complex Disease Research Studies, *BP* Blood pressure, *BHS* Bogalusa Heart Study, *BSGS* Brisbane Genetics Study, *CAAP* Coronary artery atherosclerotic plaques, *CATIS* China Antihypertensive Trial in Acute Ischemic Stroke, *CC* case-control, *CH* cohort study, *CHS* Cardiovascular Health Study, *CKB* China Kadoorie Biobank, *CS* cross-sectional, *CNTR* Chinese National Twin Registry, *DBP* Diastolic blood pressure, *DMR* Differentially Methylated Region, *FINRISK* The National FINRISK study, *FHS* Framingham Heart Study, *GENOA* Genetic Epidemiology Network of Arteriopathy, *GOLDN* Genetics of Lipid-Lowering Drugs and Diet Network Study, *HBP* High blood pressure, *HEXA* Health Examinees, *HRS* Health and Retirement Study, *HTN* hypertension, *KARE* Korea Association Resource, *KORA* Cooperative Health Research in the Region of Augsburg, *LifelinesDEEP* Lifelines Cohort Study, *LLSLFH* Leuven Longitudinal Study on Lifestyle, Fitness and Health; LBC, Lothian Birth Cohort, *LOLIPOP* London Life Sciences Population Study, *LURIC* The Ludwigshafen Risk and Cardiovascular Health Study, *MAP* Mean arterial pressure, *MEGASTROKE* Megastroke consortium; *MENA* Methyl Epigenome Network Association, *MESA* Multi-Ethnic Study of Atherosclerosis, *MR* Methylation Region, *NAS* Normative Aging Study, *NFBC1966* The Northern Finland Birth Cohort, *NT* Normotensive, *PBC* peripheral blood cells, *PP* Pulse pressure, *PURE-SA-NW* Prospective Urban and Rural Epidemiology study, *QTR* Qingdao Twin Registry, *SABRE* Southall and Brend Revisited, *SBP* Systolic blood pressure, *SMSS* Suzhou Metabolic Syndrome Study, *SYS* Saguenay Youth Study, *RS* The Rotterdam Study, *RS-BIOS* Biobank-based integrative omics study consortium, *TWB* Taiwan Biobank, *TwinsUK* Twins UK Registry, *UKB* UK Biobank, *WB* whole blood, *WBC* white blood cells, *WHI* Women’s Health Study

^1^Quality assessment determined using Newcastle-Ottawa scale. Highest score 9/9

^2^Age is either given as median (IQR), SD is unavailable, or age is not described

^3^Percentage of men not described

^4^Country not specified

**Table 4:** Associations between blood pressure or hypertension and DNA methylation age

Author, Year	Quality^1^	Population	Male (%), age (SD), sample size, country	Platform	Clock	Measure of Epigenetic Age	Definition of HTN/BP	Tissue	Associations between epigenetic age and hypertension and/or blood pressure.
Ammous et al. 2021 [22]	7/9	GENOA-AA	29, 57.1 (10.6), 1100, USA	850k	Horvath (Pan tissue), Hannum, PhenoAge, GrimAge	IEAA EEAA AA	BP: measured 3 times after 5 minute rest with the second and third averaged. HTN: SBP ≥ 140 mmHg and/or DBP ≥ 90 and/or antihypertensive medication usage.	PBC	Horvath: 1 year increase in IEAA associated with 0.37 mmHg ↑ SBP (95%Cl: 0.109, 0.627; P = 0.005), 0.16 mmHg ↑ DBP (95%Cl: 0.009, 0.303; P = 0.037), 0.23 mmHg ↑ MAP (95%Cl: 0.056, 0.393; P = 0.009) and 0.22 mmHg ↑ PP (95%Cl: 0.026, 0.414; P = 0.027). Hannum: 1 year increase in EEAA associated with 0.27 mmHg ↑ SBP (95%Cl: 0.055, 0.485; P = 0.0014) and 0.24 mmHg ↑ in PP (95%Cl: 0.082, 0.403; P = 0.003). PhenoAge: 1 year increase in AA associated with 0.22 mmHg ↑ SBP (95% Cl: 0.050, 0.395; P = 0.012), 0.12 mmHg ↑ MAP (95%Cl: 0.004, 0.229; P = 0.042) and 0.16 mmHg increase in PP (0.033, 0.293; P = 0.014). GrimAge: 1 year increase in AA associated with 0.36 mmHg ↑ SBP (95%Cl: 0.093, 0.627; P = 0.008) and 0.35 mmHg ↑ PP (95%Cl: 0.145, 0.544; P = 0.001).
Arpon et al. 2019 [202]	6/9	MENA OBEKIT + NormoP	MENA: 36.1, 47.3 (15.4), 366, Consortia OBEKIT + NormoP: 29.5, 44.8 (10.2), 268, Consortia	450 K	Horvath (Pan tissue), GrimAge	AA	BP: measured using standardised protocols.	PBC	**MENA:** Horvath: No association GrimAge: No association **OBEKIT + NormoP:** GrimAge: No association
Chen et al. 2023 [203]	7/9	HRS-VBS	HRS-VBS: 40.4, 77.0 (5.5), 1047, USA	850k	Horvath (Pan tissue), Horvath (Skin and blood), Hannum, Weidner (99 CpG), Weidner (102 CpG), VidalBralo, epiTOC, Bocklandt, Garagnami, PhenoAge, Zhang, GrimAge, DunedinPoAm38,	AA	BP: measured 3 times with 45 s intervals between with SBP variability calculated as coefficients of variation.	WB	**HRS-VBS:** Horvath: SBP variability was associated with increased AA after adjustment with multiple covariates (β = 0.084; 95% CI: 0.020, 0.147). Zhang: SBP variability was associated with increased AA after adjustment for multiple covariates (β = 0.079; 95% CI: 0.020, 0.138). PhenoAge: SBP variability was associated with increased AA after adjustment for age and sex (β = 0.100; 95% CI: 0.038, 0.161).
Chilunga et al. 2021 [24]	8/9	RODAM	Ghanian Migrants: 30, 52.4 (9.8), 347, Europe Ghanian Non-Migrants: 55, 49.9 (9.8), 365, Ghana	450k	Horvath (Pan tissue), Hannum PhenoAge GrimAge	IEAA EEAA AA	BP: measured 3 times after sitting at rest for 5 minutes.	WB	**Ghanian Migrants:** Horvath: No association. Hannum: No association. PhenoAge: No association. GrimAge: No association. **Ghanian Non-Migrants:** Horvath: No association. Hannum: No association. PhenoAge: No association. GrimAge: No association.
Dugue et al. 2018 [204]	8/9	MCCS	61, 59.0 (7.6), 2818, Australia	450k	Horvath (Pan tissue) Hannum	AA IEAA	BP: indication of measurement. HTN: self-reported antihypertensive medication usage.	BC	Horvath: No association. Hannum: No association.
Föhr et al. 2024 [74]	6/9	Discovery: FTC ERMA Replication: YFS EH-Epi	Discovery: 42.9, 52.6 (14.6), 268, Finland Replication YFS: 58.4, 43.3 (4.6), 1564, Finland EH-Epi: 44.3, 61.6 (3.7), 293, Finland	450k 850k	GrimAge DunedinPACE	AA	BP: measured repeatedly using standardised methods.	WB	Discovery GrimAge: No Association. DunedinPACE: Positive association with DBP in 2 models (P = 0.017 and P = 0.046). Positive association with DBP using within-twin-pair analysis (P = 0.005). Replication YFS GrimAge: Positive association with SBP and DBP across all models (P < 0.05). DunedinPACE: Positive association with SBP and DBP across all models (P < 0.001). EH-Epi GrimAge: No Association. DunedinPACE: Positive association with DBP after full adjustment (P < 0.05).
Gao et al. 2018 [68]	7/9	NAS	First visit: 100, 71.6 (6.5), 546, USA Second visit: 100, 75.4 (6.5), 546, USA	450k	Horvath (Pan tissue) Hannum PhenoAge	AA Δ_AA_	HTN: SBP ≥ 140 mmHg and/or DBP ≥ 90 mmHg and/or antihypertensive usage.	WB	Horvath: ↑ AA in HTN vs NTs in the first (HTN: β = 0.18 vs NT: β = 0.09; P = 0.016) and second (HTN: β = -0.01 vs β = -0.18; P = 0.016) visits. ↑ Δ_AA_ HTN vs NTs in the first (HTN: β = 0.03 vs β = -0.14; P = 0.007) and second (HTN: β = 0.01 vs β = -0.12; P = 0.007) visits. No direct association between HTN, SBP or DBP and AA at either visit. Hannum: HTN was associated with AA at the first visit (β = 2.15, P = 0.003) after adjustment for all covariates. PhenoAge: HTN was associated with AA at the first visit (β = 1.687, P = 0.002) after adjustment for all covariates. HTN was associated with AA at the second visit (β = 1.732, P = 0.003) after adjustment for age, leukocyte distribution and batch effects.
Hernández Cordero et al. 2022 [205]	5/9	St Paul’s Hospital HIV Bronchoscopy cohort, START	Broncho: 78, 56 (52–63)^2^, 18, Canada START: 90.3, 40 (34–49)^2^, 378, International	850k	GrimAge	AA	BP: indication of measurement HTN: SBP ≥ 140 mmHg and/or DBP ≥ 90 mmHg and/or antihypertensive medication usage	Broncho: airway epithelial brushings START: WB	**Broncho:** No association with HTN or BP performed. **START:** GrimAge: ↑ AA was associated with HTN (P = 0.010).
Horvath et al. 2016 [23]	8/9	BHS, WHI	WHI: 0, 50–79^2^, 1462, USA BHS: N/A^3^, 28–51.3^2^, 969, USA	450k	Horvath (Pan tissue), Hannum	AA IEAA EEAA	HTN: SBP ≥ 140 mmHg and/or DBP ≥ 90 mmHg and/or antihypertensive medication usage and/or HTN diagnosis	WHI: WBC BHS: WB	**WHI:** Horvath: No association. Hannum: No association. **BHS:** Horvath: ↑ IEAA in HTN vs Controls in the total cohort (P = 0.0035) and in African Americans (P = 0.00049) Hannum: HTN was associated with EEAA (β = 1.25, P = 1.7E-05). ↑ EEAA in HTN vs Controls in the total cohort (P = 8E-05) and when stratified by race (Caucasian: P = 0.00039; African American: P = 0.0025).
Horvath et al. 2022 [206]	6/9	NNAB, MHBB, NNTC	N/A^3^, 27.9–91.2^2^, 133 (75 infected with HIV), USA	450k	Horvath (Pan tissue), Horvath (Skin and blood)	AA	HTN: self-reported and/or medical record review.	Adipose, WB, Bone, marrow, Heart, Kidney, Liver, Lung, Lymph Node, Muscle, Spleen, Pituitary gland	**NNAB, MHBB, NNTC combined:** Horvath (Pan tissue): ↑ AA in HTN vs NT in all tissues combined (P = 4.5E-05), in kidney (P = 0.0048), liver (P = 0.028) and lymph nodes (P = 0.033). Horvath (Skin and Blood): ↑ AA in HTN vs NT in all tissues combined (P = 0.00036) and kidney (P = 0.0095).
Irvin et al 2018 [25]	7/9	GOLDN	42, 46.6–50.7^2^, 993, USA	450k	Horvath (Pan tissue) Hannum	IEAA EEAA	HTN: SBP ≥ 140 mmHg and/or DBP ≥ 90 mmHg and/or antihypertensive medication usage.	CD4 + T Cells	Horvath: No association. Hannum: ↑ prevalence of HTN was associated with above the median EEAA (P = 0.0001).
Jiang et al. 2022 [75]	8/9	CATHGEN	58.4, 60.1 (12.4), 562, USA	850k	Horvath (Pan tissue) PhenoAge, GrimAge	AA	BP: taken from medical records. HTN: medical history.	WB	Horvath: Positive association between AA and DBP (P = 0.007). PhenoAge: No association. GrimAge: Negative association between AA and SBP (P = 0.002).
Kresovich et al. 2023 [82]	6/9	Sister Study	0, 56 (9.0), 4419, USA and Puerto Rico	450 K	PhenoAge GrimAge DunedinPACE	AA	BP: measured in a sitting position 3 times 2 min apart. HTN: SBP ≥ 140 mmHg and/or DBP ≥ 90 mmHg and/or antihypertensive medication usage.	WB	PhenoAge: Positive association with HTN (OR: 1.16, 95%CI: 1.05-1.28). GrimAge: Positive association with HTN (OR :1.28, 95%CI: 1.14, 1.45). DunedinPACE: Positive association with HTN (OR: 1.16, 95%CI: 1.03, 1.30). Normotensive women at baseline with higher AA were more likely to be diagnosed with incident HTN for PhenoAge (HR: 1.09, 95%CI: 0.97, 1.23), GrimAge (HR: 1.16, 95%CI: 0.99, 1.36) and DunedinPACE (HR: 1.16, 95%CI: 1.01, 1.33).
Levine et al. 2018 [18]	8/9	WHI	0, 50–79^2^, 4771, USA	450k	PhenoAge	AA	BP: measured seated after 5 min rest. Average taken. HTN: SBP ≥ 140 mmHg and/or DBP ≥ 90 mmHg and/or antihypertensive medication usage.	WBC	PhenoAge: Positive association between AA and SBP (Bicor= 0.08, P = 1E-06).
Li et al. 2019 [207]	7/9	InterGEN	0, 31.7 (5.7), 232, USA	850k	Horvath (Pan tissue)	AA	HTN: self-reported.	Saliva	Horvath: 1.49-year ↑ AA in HTN vs NT individuals (β: 1.49; 95% Cl: 0.11, 2.86).
Lin et al. 2023 [208]	5/9	TWB	50.2, 50.3 (11.3), 2474, Taiwan	850 K	Horvath (Pan tissue) Hannum PhenoAge GrimAge DunedinPACE	AA	HTN: SBP ≥ 130 mmHg and/or DBP ≥ 80 mmHg and/or antihypertensive medication usage	WB	No association (P < 4E-04).
Lind et al. 2018 [69]	6/9	Gen Pop	N/A^3^, 70.2 (0.2), 967, Sweden	450k	Horvath (Pan tissue) Hannum	DiffAge	BP: measured 3 times after 30 minutes rest.	WB	Horvath: Positive association between DiffAge and SBP (β = 0.02; 95%Cl: 0, 0.03; P = 0.012).
Lo et al. 2022 [209]	6/9	TWB	50.2, 49.8 (11.1), 2474, Taiwan	850k	Horvath (Pan tissue), Hannum, PhenoAge, GrimAge	IEAA, AA	HTN: SBP ≥ 140 mmHg and/or DBP ≥ 90 mmHg and/or self-reported history of HTN. Ideal BP: SBP ≤ 120 mmHg and/or DBP ≤ 80 mmHg.	WB	Horvath: More participants with ideal BP in the lowest tertile of IEAA (P < 0.001). Hannum: More participants with idea BP in lowest tertile of AA (P < 0001). PhenoAge: More participants with ideal BP in the lowest tertile of AA (P < 0.01). GrimAge: More participants with ideal BP in the lowest tertile of AA (P < 0.001).
Lu et al. 2019 [19]	7/9	FHS WHI JHS InCHIANTI	FHS: 47, 66.9 (8.6), 625, USA WHI BA23: 0, 65.1 (7.1), 2107, USA WHI EMPC: 0, 63.3 (7.0), 1972, USA JHS: 37, 56.2 (12.3), 1747 USA InCHIANTI: 46, 67.0 (16.6), Italy	450k	GrimAge	AA	HTN: SBP ≥ 140 mmHg and/or DBP ≥ 90 mmHg and/or antihypertensive medication usage.	FHS: BC WHI: WB InCHIANTI: BC JHS: PBC	GrimAge: ↑ AA was associated with HTN (OR = 1.04, P = 5.1E-13). ↑ AA was associated with SBP (bicor = 0.07; P = 9E-07).
Marinello et al. 2024 [210]	4/9	SPHERE	24.4, 51.7 (18.1), 190, Italy	MSP	Zbieć-Piekarska	AA	BP: indication of measurements HTN: use of antihypertensive medications	BC	Zbieć-Piekarska: Positive association between SBP and AA (β = 0.045, P = 0.019).
Marioni et al. 2015 [20]	8/9	LBC1921 LBC1936 FHS NAS	LBC1921: 40, 79.1 (0.6), 446, Scotland LBC1936: 51, 69.5 (0.8), 920, Scotland FHS: 46, 66.3 (8.9), 2635, USA NAS: 100, 72.9 (6.9), 657, USA	450k	Horvath (Pan tissue), Hannum	Δ_Age_	**LBC:** HBP**:** self-reported yes/no **FHS and NAS:** HTN: SBP ≥ 140 mmHg and/or DBP ≥ 90 mmHg and/or antihypertensive medication usage.	LBC1921: WB LBC1936: WB FHS: PBC NAS: BC	**LBC1921:** Horvath: No association. Hannum: No association. **LBC1936:** Horvath: No association. Hannum: No association. **FHS:** Horvath: Negative association between Horvath Δ_Age_ and HBP (β = -0.22, P = 8.6E-07). Hannum: No association. **NAS:** Horvath: No association. Hannum: No association.
McCartney et al. 2018 [80]	8/9	Generation Scotland	37.6, 48.51 (13.99), 5101, Scotland	850k	Horvath (Pan tissue), Hannum	IEAA EEAA	HBP: self-reported BP yes/no	PBC	Horvath: No association. Hannum: Positive association between EEAA and HBP (β = 0.177; 95%Cl: 0.064, 0.29; P = 0.002).
Perez et al. 2022 [211]	6/9	InterGEN	0, 31.2 (27.3–35.6)^2^, 227, USA	850k	Horvath (Pan tissue)	AA	HTN: self-reported.	WB	Horvath: Positive association between AA and HTN (β = 0.08; 95%Cl: 1.01, 1.17).
Philibert et al. 2024 [212]	5/9	ProSAAF	41, 49.6 (9.3), 278, USA	850 K	GrimAge DunedinPACE	AA	HTN: self-reported	WB	GrimAge: Positive correlation with HTN (P < 0.05) DundinPACE: Positive correlation with HTN (P < 0.05).
Pottinger et al. 2021 [85]	7/9	WHI	0, 64.2 (7.1), 2170, USA	450k	Horvath (Pan tissue) Hannum	IEAA EEAA	HTN: SBP ≥ 140 mmHg and/or DBP ≥ 90 mmHg and/or antihypertensive medication usage	WB	Horvath: No association. Hannum: No association.
Qin et al. 2021 [213]	7/9	SJLIFE	47, 31.6 (6.0, 66.4)^2^, 2139, USA	850k	PhenoAge	AA	HTN: SBP ≥ 140 mmHg and/or DBP ≥ 90 mmHg and/or antihypertensive medication usage	WB	PhenoAge: HTN was associated with EAA in time to event analysis (third vs first tertile, relative rate [RR] = 1.83; 95%CI: 1.17, 2.83; P_trend_ = 0.005)
Quach et al. 2017 [70]	7/9	WHI InCHIANTI	WHI: 0, 64 (7.1), 4173, USA InCHIANTI: 44, 71 (16), 402, Italy	450k	Horvath (Pan tissue) Hannum	IEAA EEAA	HTN: SBP ≥ 140 mmHg and/or DBP ≥ 90 mmHg and/or antihypertensive medication usage. BP: measured seated after 5 min rest. Average taken.	WB	Horvath: ↑ IEAA correlated with SBP (bicor=0.04, P = 5E-03) and DBP (bicor=0.05, P = 3E-03). Hannum: ↑ EEAA correlated with SBP (bicor=0.07, P = 4E-06) and DBP (bicor=0.04, P = 0.01).
Roberts et al. 2021 [72]	8/9	ARIC CHS FHS	ARIC: 36.1, 56.5 (5.8), 2159, USA CHS: 38.4, 74.1 (5.2), 719, USA FHS: 44.4, 65.8 (8.8), 2362, USA	450k	Horvath, Hannum, PAI-1, PhenoAge, GrimAge	AA	HTN: SBP ≥ 140 mmHg and/or DBP ≥ 90 mmHg and/or antihypertensive medication usage BP: measured seated after 5 min rest. Average taken.	WB	Horvath: Positive association between AA and anti-HTN medication usage (OR = 1.07; 95%Cl: 1.02, 1.12; P = 0.0011). Hannum: Positive association between AA and SBP (β = 0.76; 95%Cl: 0.28, 1.24; P = 0.002) and anti-HTN medication usage (OR = 1.11; 95%Cl: 1.04, 1.18; P = 0.0011). PhenoAge: Positive association between AA and SBP (β = 0.71; 95%Cl: 0.34, 1.09; P = 0.0002) and anti-HTN medication usage (OR = 1.11; 95%Cl: 1.02, 1.22; P = 0.0157). PAI-1: Positive association between AA and SBP (β = 1.67; 95%Cl: 1.15, 2.19; P < 0.0001) and anti-HTN medication usage (β = 1.44; 95%Cl: 1.16, 1.77; P = 0.0008). GrimAge: Positive association between AA and SBP (β = 1.07; 95%Cl: 0.36, 1.77; P = 0.0031). Negative association with DBP (OR = -0.50; 95%Cl: -0.92, -0.08; P = 0.0206).
Robinson et al. 2020 [77]	8/9	Airwave	60.5, 41.24 (9.1), 2238, UK	850 K	Horvath Hannum PhenoAge	AA	HTN: SBP ≥ 140 mmHg and/or DBP ≥ 90 mmHg and/or antihypertensive medication usage	WB	Horvath: No association. Hannum: No association. PhenoAge: Positive association between AA and HTN (β = 1.15; 95%Cl: 0.46, 1.84; P = 0.0012).
Roetker et al. 2018 [78]	7/9	ARIC	35, 57 (6), 2543, USA	450k	Horvath (Pan tissue), Hannum	AA	HTN: SBP ≥ 140 mmHg and/or DBP ≥ 90 mmHg and/or antihypertensive medication usage	WBC	Horvath: No association. Hannum: Positive association between AA and anti-HTN use (β = 0.47; P = 0.04).
Smith et al. 2019 [73]	8/9	GENOA	28.9, 58.0 (10.1), 1390, USA	850k	Horvath (Pan tissue) Hannum	IEAA EEAA	BP: BP was measured twice seated after 5 minutes, average taken. HTN: SBP ≥ 140 mmHg and/or DBP ≥ 90 and/or antihypertensive medication usage	PBC	Horvath: No association. Hannum: Positive association between EEAA and SBP when adjusting for antihypertensive medication (β = 0.188; P = 0.015).
Tamman et al. 2019 [67]	6/9	NHRVS	100, 63.1 (14.0), 1135, USA	850k	Horvath (Pan tissue)	Δ_Age_	HTN: self-reported.	Saliva	Horvath: ↑ HTN prevalence as associated with ↑ odds of Δ_Age_ (OR: 1.48, 95%Cl: 1.02-2.15).
Tang et al. 2023 [66]	5/9	SATSA	58.8, 66.6 (7.6) 672, Sweden	450 K	Horvath (Pan tissue) Hannum PhenoAge GrimAge	PCAge	HTN: SBP ≥ 140 mmHg and/or DBP ≥ 90 and/or antihypertensive medication usage	WBC	GrimAge: Negative association between anti-HTN usage and PCGrimAge (β = -0.39; 95% CI: -0.67, -0.12).
Wan et al. 2024 [65]	6/9	HRS	42.1, 67.3 (9.5), 3708, USA	850 K	GrimAge	DiffAge	HTN: self reported (yes/no).	WB	GrimAge: ↑ GrimAge in participants with HTN and cognition (β = 0.963; 95% CI: 0.711, 1.214).
Wang et al. 2023 [169]	7/9	FHS	53.8, 58.1 (24, 94) 3823, USA	450 K	PhenoAge GrimAge	AA	HTN: SBP ≥ 130 mmHg and/or DBP ≥ 80 mmHg and/or antihypertensive medication usage	WB	PhenoAge: Positive association between AA and HTN (OR: 1.09, 95%Cl: 1.05-1.12). GrimAge: Positive association between AA and HTN (OR: 1.05, 95%Cl: 1.03-1.08).
Xia et al. 2024 [76]	6/9	SCS	52.6, 61.5 (8.4), 95, China	850 K	Horvath (Pan tissue)	AA	BP: indication of measurement HTN: ≥140 mmHg and/or ≥90 mmHg and/or antihypertensive medication usage.	WB	Horvath: ↑ DBP in individuals with AA compared to non-AA individuals (P < 0.007). No association with SBP or HTN.
Xiao et al. 2022 [71]	7/9	Gen pop	42.9, 78.5 (16.1), 280, China	850k	Horvath (Pan tissue), Hannum	Δ_Age_, Aging rate	BP: measured 2-3 times seated at rest for 5 minutes. HTN: ≥140 mmHg and/or ≥90 mmHg and/or antihypertensive medication usage.	WBC	Horvath: 10 mmHg increment of SBP was associated with ↑ 0.608 years of Δ_Age_ (95%Cl: 0.23, 0.98; P = 0.002) and ↑ 0.007 increase in aging rate (95%Cl: 0.002, 0.012; 0.004). 10 mmHg increment of PP was associated with ↑ 1.12 years of Δ_Age_ (95%Cl: 0.63, 1.61; P < 0.001) and ↑ 0.013 in aging rate (95%Cl: 0.007, 0.020; P < 0.001). Positive association between HTN and Δ_Age_ (β = 3.10; 95%Cl: 1.07, 5.13; P = 0.003) and aging rate (β = 0.04; 95%Cl: 0.015, 0.065; P = 0.002). Hannum: 10 mmHg increment of SBP was associated with ↑ 0.613 year of Δ_Age_ (95%Cl: 0.28, 1.08; P = 0.001) and ↑ 0.009 in aging rate (95%Cl: 0.003, 0.014; P = 0.003). 10 mmHg increment of PP was associated with ↑1.22 years of Δ_Age_ (95%Cl: 0.70, 1.75; P < 001) and ↑ 0.016 in aging rate (95%Cl: 0.008, 0.023; P < 001).

*AA* age acceleration, *ARIC* Atherosclerosis Risk in Communities, *BC* buffy coat, *BHS* Bogalusa Heart Study, *BP* blood pressure, *CATHGEN* Catheterisation Genetics, *CHARLS* China Health and Retirement Longitudinal Study, *CHS* Cardiovascular Health Study, *DBP* diastolic blood pressure, *EEAA* extrinsic epigenetic age acceleration, *EH-epi* Essential Hypertension Epigenetics Study, *ERMA* Estrogenic Regulation of Muscle Apoptosis, *FHS* Framingham Heart Study, *FTC* Finnish Twin Cohort, *GENOA-AA* Genetic Epidemiology Network of Arteriopathy – African Americans, *Gen pop* general population, *GOLDN* Genetics of Lipid-Lowering Drugs and Diet Network Study, *HBP* high blood pressure, *HTN* hypertension, *HRS-VBS* Health and Retirement Study – Venous Blood Study, *IEAA* intrinsic epigenetic age acceleration, *InCHIANTI* Invecchaire in Chianti, *InterGen* Intergenerational Impact of Genetic and Psychological Factors on Blood Pressure, *JHS* Jackson Heart Study, *LBC1921* Lothian Birth Cohort (1921), *LBC1936* Lothian Birth Cohort (1936), *MAP* mean arterial pressure, *MENA* Methyl Epigenome Network Association, *MCCS* Melbourne Collaborative Cohort Study, *MHBB* Manhattan HIV Brain Bank, *NAS* Normative Aging Study, *NHANES* National Health and Nutrition Examination Survey, *NHRVS* National Health and Resilience in Veterans Study, *NNAB* National Neurological AIDS Bank, *NNTC* National NeuroAIDS Tissue Consortium, *OBEKIT+NormoP* Development of a Nutrigenetic Test for Personalized Prescription of Body Weight Loss Diet, *PBC* peripheral blood cells, *PP* pulse pressure, *ProSAAF* Promoting Strong African American Families, *RODAM* Research on Obesity and Diabetes among African Migrants, *SATSA* Swedish Adoption/Twin Study of Aging, *SBP* systolic blood pressure, *SCS* Shanghai Changfeng Study, *SJLIFE* St. Jude Lifetime Cohort Study, *SPHERE* Secondary prevention of heart disease in general practice, *START* Strategic Timing of Antiretroviral Treatment, *TWB* Taiwan Biobank, *WB* whole blood, *WBC* white blood cell, *WHI* Women’s Health Initiative, *YFS* Young Finns Study

^a^Quality assessment determined using Newcastle-Ottawa scale. Highest score 9/9

^b^Age is either given as median (IQR), SD is unavailable, or age is not described

^c^Percentage of men not described, or not available

### Outcome assessment

Studies were included if they investigated an association between DNA methylation and either HTN or BP, resulting in outcomes being reported separately as either a diagnosis of HTN (38.8%), a measure of BP (34.8%), or both (26.4%) (Tables 1–4). HTN was defined by European guidelines in 64.7% of 116 studies reporting HTN, while 9.5% of studies defined HTN according to American guidelines. In addition, 8.6% of studies reported HTN based on medical history, a further 9.5% based on self-reported HTN, and 0.9% defined HTN as SBP ≥ 160 mmHg and/or DBP ≥ 100 mmHg. Furthermore, 0.9% of studies reported HTN as a binary yes/no answer without further definition, while another 0.9% of studies did not indicate HTN definition and 1.7% of studies reported HTN as solely antihypertensive medication usage. Additionally, 3.4% of studies reported multiple definitions of HTN. Regarding BP measurement, 61.5% of 109 studies reporting BP measured BP in a standardised manner (after a period of rest, seated with multiple measurements taken), 6.4% of studies reported BP levels from medical history, 0.9% measured BP over a 24 h period, 2.8% report multiple BP measurement methods and 28.4% provided an indication of BP measurement without further definition.

### Risk of Bias assessment

A case-control design was used in 32.1% of studies, while the majority of studies (55.8%) used a cross-sectional approach. Most of the included studies (54.5%) achieved a moderate rating of 5-6 stars, suggesting moderate methodological quality. A substantial number of studies (43.0%) attained a higher rating of 7-9 stars, indicating good methodological quality, while only 2.4% of studies were rated below 4 stars, indicating lower quality (Table S3).

### Global methylation

Global DNA methylation was investigated in 25 publications, including 4 studies within large prospective cohorts and 21 original study cohorts (Table 1). Global DNA methylation analysis was performed in healthy individuals in 16.0% of studies and was investigated in several disease state cohorts, such as HTN (20.0% of studies) and diabetes (16.0% of studies). The majority of studies investigating global methylation, focused on repeat sequences and transposable elements as a proxy, which have been shown to correlate with total genomic content [40, 41]. The remaining studies investigating global methylation assessed DNA methylation as a level of 5 mC or as a percentage of total cytosine (MC/C ratio).

Several studies report conflicting findings in regard to the association between SBP and *LINE-1* methylation, with varying directions of association (Fig. 2B) [42, 43]. Three studies report a negative association between DBP and *LINE-1* methylation [26, 43, 44]. Furthermore, a single study also reports a negative association between HTN and *LINE-1* methylation [45].

*Alu* methylation was associated with both SBP and DBP with negative and positive associations [26, 46, 47]. *Alu* methylation was negatively associated with HTN [47] and positively associated with pre-HTN [48]. Additionally, 5mC was negatively associated with HTN [49, 50]. No significant associations were reported between BP traits and MC/C ratio.

### Gene-specific DNA methylation

Gene-specific DNA methylation of 138 candidate genes was examined utilising data from 81 distinct studies (Table 2). Significant associations with either BP or HTN were reported for a total of 88 candidate genes. Most studies were conducted in original cohorts comprising individuals with diverse characteristics, including healthy individuals (9.9% of studies), those with HTN (38.3%), and obese individuals (6.2%), in addition to other disease states.

Twenty-five studies reported associations between SBP and DNA methylation examining a total of 45 genes. The most commonly reported genes included Tumour Necrosis Factor Alpha *(TNF-a)*, Glucocorticoid receptor gene *(GR)* and Interleukin-6 (*IL-6)*, all of which demonstrated conflicting directions of association between methylation and SBP (Fig. 2C) [51–54]. Similarly, among the 26 studies reporting an association between DNA methylation and DBP, the most commonly reported genes also included *TNF-a*, *GR* and *IL-6*, along with Toll-like receptor 2 (*TLR2*) methylation, which exhibited both positive and negative associations with DBP (Fig. 2b) [54, 55].

Angiotensin II Receptor Type 1 (*AGTR1*) methylation was the most frequently studied gene in relation to HTN, with studies again reporting both positive and negative associations (Fig. 2C) [56–58]. Cystathionine Beta Synthase (*CBS*) methylation was positively associated with HTN [59, 60], while *ADD1* methylation was negatively associated with HTN [61, 62]. *MTHFR* methylation was positively associated with HTN, in addition to other genes involved in folate and one-carbon metabolism, such as dihydrofolate reductase (*DHFR*) and methylenetetrahydrofolate dehydrogenase (*MTHFD1*) [29, 63, 64].

### Epigenome-wide methylation

**Fig. 3:**
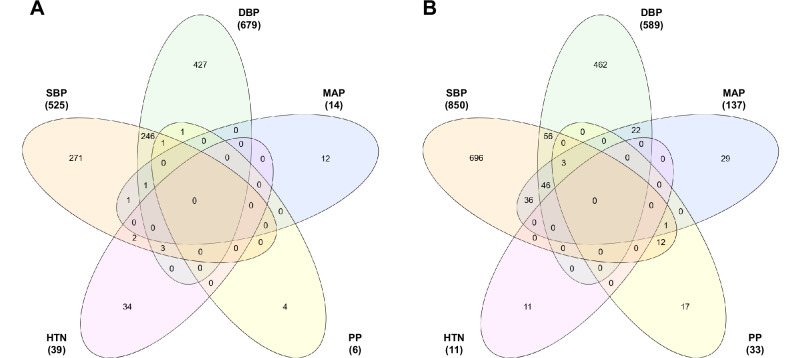
**A** CpG sites and (**B**) Differentially methylated regions (DMRs) associated with blood pressure (BP) traits. Systolic blood pressure (SBP), Diastolic blood pressure (DBP), Hypertension (HTN), Pulse Pressure (PP), Mean arterial pressure (MAP)

Epigenome-wide methylation was investigated in 35 studies, resulting in the identification of 1003 CpG sites associated with various BP traits (Fig. 3A). The most commonly reported CpG sites included cg19693031 and cg18120259 which were reported in 5 publications (Table S4). The top genes annotated to these CpGs included Thioredoxin Interaction Protein (*TXNIP*) and a long intergenic non-protein coding RNA (*LOC100132354*). In total, CpG sites were annotated to 569 genes, with 61 genes reported in >1 publication. 1391 DMRs were associated with various BP traits (Fig. 3B). Eight DMRs were reported in >1 publication (Table S5).

**Fig. 4:**
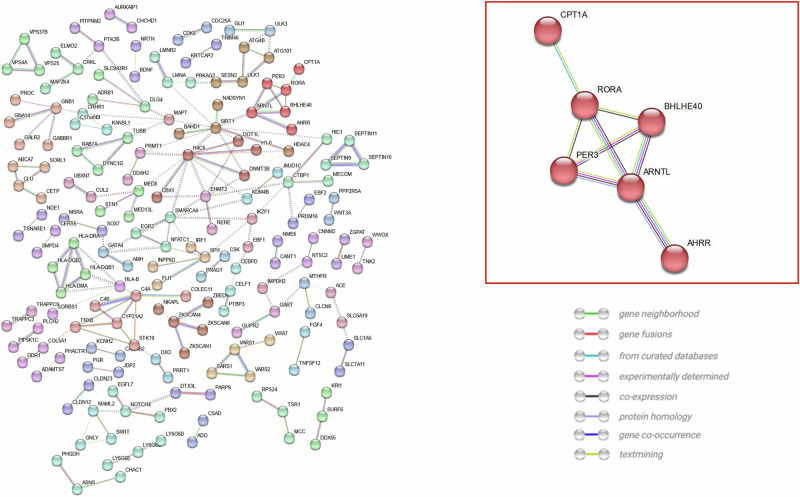
Network analysis of genes annotated to CpG sites associated with any BP outcome using STRING-db; genes without connections are hidden

Functional enrichment analysis of genes annotated from CpG sites significantly associated with any BP trait indicated several highly enriched disease terms, including systemic lupus erythematosus (P_FDR_ = 1.84E-17), schizophrenia (P_FDR_ = 0.001), HTN (P_FDR_ = 0.002), and SBP (P_FDR_ = 0.005) (Table S6). Functional enrichment of the genes annotated from DMRs significantly associated with any BP trait identified biological processes such as regulation of transcription by RNA polymerase II (P_FDR_ = 0.0003), cartilage development (P_FDR_ = 0.0004) and intracellular signal transduction (P_FDR_ = 0.009) (Table S7). Regarding cellular components, significantly associated terms included chromatin (P_FDR_ = 1.59E-07), nucleoplasm (P_FDR_ = 0.0002), post synaptic density (P_FDR_ = 0.003), cell surface (P_FDR_ = 0.032), focal adhesion (P_FDR_ = 0.032), synapse (P_FDR_ = 0.049), and axon (P_FDR_ = 0.049). (Table S7). In terms of molecular function, protein binding was found to be statistically significant (P_FDR_ = 0.0001), in addition to RNA polymerase II-specific DNA-binding transcription factor activity (P_FDR_ = 0.0003), metal ion binding (P_FDR_ = 0.0003), zinc ion binding (P_FDR_ = 0.011), sequence-specific double-stranded DNA binding (P_FDR_ = 0.013), and identical protein binding (P_FDR_ = 0.034) (Table S7). STRING analysis with the confidence setting ‘high’ revealed significant evidence for protein-protein interactions between the products of genes annotated to CpG sites associated with BP (P = 1.46E-07) (Fig. 4). The average node degree was 0.83, while the average local clustering coefficient was 0.27.

### Epigenetic age

Fourteen DNA methylation algorithms were implemented across 37 studies investigating epigenetic age (Table 4). Most studies calculated epigenetic age using more than one algorithm, with the Horvath (Pan-tissue) clock being the most frequently used (73.0% of studies). Epigenetic age was most commonly reported as EAA; however, 6 studies additionally reported Delta Age (ΔAge), DiffAge, PCAge, changing rate of age acceleration (ΔAA), and ageing rate [20, 65–69]. The majority of studies within this review demonstrate a very high correlation between epigenetic age and chronological age (r = 0.7–0.9), which is representative of the function of these algorithms, however, some individual studies did report lower correlations between epigenetic and chronological age [70].

Six studies reported positive associations between AgeAccelHorvath and SBP [22, 69–71]. Four studies reported positive associations between AgeAccelHannum and SBP [22, 70, 72, 73]. Similarly, 4 studies reported a positive association between PhenoAgeAccel and SBP [18, 22, 72, 73]. GrimAgeAccel was positively associated with SBP in 4 separate studies, while a further study reported a negative association [19, 20, 72, 74, 75].

Four studies reported a positive association between AgeAccelHorvath and DBP [20, 70, 75, 76]. AgeAccelHannum was associated with DBP in 1 study [70], while a positive association between GrimAgeAccel and DBP was reported in 2 studies [72, 74]. No significant associations were reported between DBP and PhenoAgeAccel.

### Meta-analysis

A meta-analysis was conducted to examine the association between HTN and EAA. Data from a total of 16,136 individuals across 8 studies were included in the analysis. Beta coefficients and standard errors (or calculated standard errors from published 95% confidence intervals) were obtained from each study. The majority of studies assessed DNA methylation in blood, while 1 study used saliva.

**Fig. 5:**
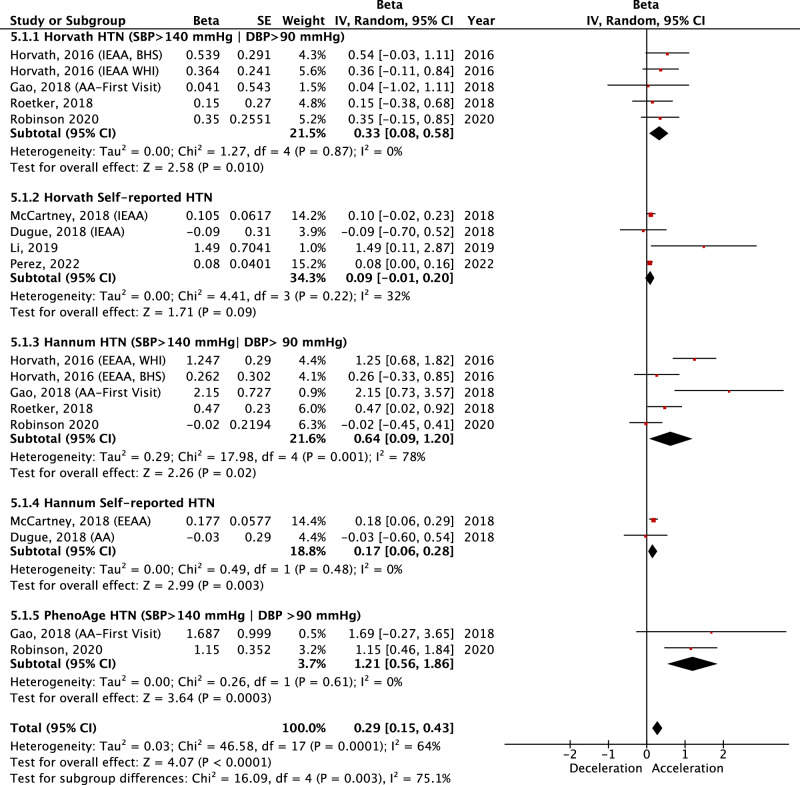
Random effects meta-analysis of the association of HTN with epigenetic age acceleration subgrouped by epigenetic clock algorithm and clinically-defined v self-report of hypertension. Horizontal lines represent the 95% confidence interval (CI) for each study. Diamonds indicate pooled effect and 95%CI for each subgroup and overall effect (Z). χ ^2^ chi-squared test assesses whether observed difference in results are compatible with chance alone; I^2^ heterogeneity index (0%–100%); SD, standard deviation; IV, Random, a random effects meta-analysis is applied with weights based on inverse variances

The meta-analysis, using a random-effects model (Fig. 5), demonstrated a significant positive association between HTN and EAA across the three epigenetic clock algorithms (*β* = 0.29, *P* < 0.001; 95% Cl: 0.15–0.43, *P* < 0.0001). Subgroup analysis further revealed that clinically measured HTN as determined by European guidelines [31], was associated with each epigenetic clock individually (Horvath: β = 0.33, 95% Cl: 0.08–0.58, *P* = 0.010; Hannum: β = 0.64, 95% Cl: 0.09–1.20, *P* = 0.02; PhenoAge: β = 1.21, 95% Cl: 0.56–1.86, *P* = 0.0003). Further subgroup analysis based on self-reported HTN status demonstrated no significant association with AgeAccelHorvath (β = 0.09, *P* = 0.09, 95% Cl: −0.01 to 0.20); however, a significant association with AgeAccelHannum (β = 0.17, *P* = 0.003, 95% Cl: 0.06–0.28) was observed. Heterogeneity was observed in the overall meta-analysis across the three epigenetic clock algorithms (I^2^ = 64%, *P* = 0.001) and for AgeAccelHannum in individuals who reported HTN according to European guidelines (I^2^ = 78%, *P* = 0.001). No significant heterogeneity was observed in either the AgeAccelHorvath subgroup, when HTN was either clinically measured or self-reported (I^2^ = 0%, *P* = 0.87; I^2^ = 32%, *P* = 0.22), or the AgeAccelHannum subgroup when HTN was self-reported (I^2^ = 0%, *P* = 0.48), or PhenoAgeAccel (I^2^ = 0%, *P* = 0.61) when HTN was clinically measured.

### Publication bias and Sensitivity analysis

Visual assessment of publication bias by funnel plot (Figure S1) determined significant asymmetry which was further confirmed by a significant Egger’s regression test (*P* = 0.0002). The small number of studies included in the meta-analysis or heterogeneity among these studies may have influenced the results of Egger’s regression test and observed asymmetry, introducing potential variability and making limiting the ability to draw definitive conclusions regarding publication bias. Trim-and-fill analysis estimated three missing studies (Figure S2), with the overall association measure based on this analysis remaining significant (β = 0.260, *P* = 0.0005; I^2^ = 65.18%, *P* < 0.0001). Given the observed heterogeneity, we performed a sensitivity analysis of the eight included studies. The overall effect remained consistent overall effect after sequential exclusion of each individual study, (Figure S3) indicating that the findings are stable and not driven by one particular study.

## Discussion

This study is the first to demonstrate that HTN is significantly associated with accelerated epigenetic age by systematically evaluating current evidence, highlighting an important role for DNA methylation in the development and pathophysiology of HTN in adults. Furthermore, each epigenetic clock algorithm individually demonstrated that clinically measured HTN was significantly associated with increased EAA.

In our meta-analysis of 16,136 individuals, we demonstrated a significant association between HTN and increased EAA combining three main epigenetic clock algorithms. Accelerated epigenetic ageing was observed in individuals with clinically measured HTN in subgroup analysis using the Horvath clock, despite none of the included studies individually reaching statistical significance [17, 18, 68, 77, 78], perhaps due to a lack of statistical power within individual studies. Similarly, a small meta-analysis using the Horvath clock previously reported accelerated biological ageing in >5600 participants with clinically defined HTN across three pooled studies in which no significant effect was observed in each individual cohort [72]. While results are generally consistent for clinically measured hypertension, there appears to be a disparity in using self-reported HTN which may prevent identification of positive associations between epigenetic age and HTN, introducing potential misclassification bias. Self-reporting is considerably reliable for ruling out HTN, however, the probability of correctly identifying patients with HTN through self-reporting is only mildly sensitive, correctly identifying individuals with HTN in approximately 37% of cases, indicating that a large number of hypertensive individuals remain undiagnosed [79]. In accordance, one well-powered study within our meta-analysis reported no significant association between Horvath EAA and self-reported HTN in >5100 participants; perhaps attributable to the use of self-reported HTN rather than more discriminatory clinical measurement [80]. Subgroup analysis, employing Hannum and PhenoAge clocks, also demonstrated accelerated epigenetic ageing in individuals with both clinically and self-reported HTN despite a lack of consistent evidence across individual studies. Increased EAA was also consistently associated with BP traits, such as SBP and DBP, within studies that were ineligible for meta-analysis (Table 4).

The overall association between HTN and EAA was observed despite each clock using different CpG sites and parameters in the algorithms used to calculate epigenetic age, therefore, it is not surprising that heterogeneity was observed in the meta-analysis. The heterogeneity observed in the overall meta-analysis may reflect differences in study design across the limited number of studies, including variation in study design, epigenetic clock algorithm, participant characteristics and levels of covariate adjustment. The observed heterogeneity likely reflects the biological and methodological diversity inherent to epigenetic ageing measures. The pooled estimate is therefore interpreted with caution, as an overall summary of the association between EAA and HTN rather than a precise effect size. Although the meta-analysis was limited by the small number of studies, the overall positive effect of the meta-analysis remained robust following sensitivity analysis indicating that the pooled result is not driven by one study (Figure S3). Lack of overlap in genomic locations used in epigenetic clock algorithms suggests that each clock investigates a separate measure of biological age, drawing methylation markers from entirely different regions of the genome [81]. Notably GrimAgeAccel was not represented among eligible studies for meta-analysis, however, the overall association of HTN and EAA using three first- and second- generation clocks, suggests wide-ranging perturbations across the epigenome.

The causal relationship between EAA and hypertension remains an area of active investigation. Longitudinal studies have suggested that individuals with higher baseline EAA are at increased risk of developing incident hypertension [82], supporting the hypothesis that accelerated epigenetic aging may predispose to hypertension. Conversely, other evidence indicates that established hypertension may itself contribute to EAA through mechanisms such as vascular remodelling, chronic low-grade inflammation, oxidative stress, and endothelial injury, which are known to influence DNA methylation patterns [68, 78]. These mechanisms highlight the potential for a feedback loop where increasing EAA and HTN mutually reinforce each other, and it currently remains unclear whether epigenetic alterations precede adverse cardiovascular remodelling events in HTN [83]. It is plausible that epigenetic age acceleration is correlated with HTN but may not lie along the causal pathway of incident HTN. Future research directed towards dissecting molecular mechanisms connecting EAA and HTN may help elucidate the mechanism underlying hypertension physiology. Furthermore, studies investigating whether epigenetic aging influences gene expression at the transcriptomic or proteomic level and development of models to identify key physiological pathways in HTN will be valuable in answering these important questions.

There is currently immense interest in epigenome-wide methylation and epigenetic age investigations. In the systematic review a diverse range of experimental design was observed in included studies (Fig. 2A). Large cohorts such as the Women’s Health Initiative (WHI) have employed the 450 K array which has proved very useful and has been extensively used over the past decade [18, 19, 23, 70, 84, 85]. Moving forward studies should take advantage of assays providing more epigenomic coverage e.g., the Generation Scotland Family Health Study utilises the IlluminaMethylationEPIC array which covers over 850 CpG sites [80, 86, 87]. DNA repetitive element methylation, however, is not directly analysed in commonly used methylation arrays and are therefore reported separately. Repeat elements *LINE-1* and *Alu* were the most commonly reported markers of global methylation within this review (Table 1). Global methylation is inversely associated with BP traits in the majority of studies reporting significant outcomes (Fig. 2B) consistent with previous findings [8, 10].

The most frequently reported candidate gene was *AGTR1* (Fig. 2C), methylation of which has been inversely associated with HTN [88], while SNPs in *AGTR1* result in decreased expression and increased HTN risk [89]. *MTHFR* methylation was also reported to be associated with HTN (Fig. 2C). Common polymorphisms in *MTHFR*, resulting in the 677TT genotype yields an increased risk of HTN [90]. Hypermethylation of *MTHFR* is observed in individuals with the TT genotype, blood pressure is also higher compared to CC counterparts. Significant reduction in *MTHFR* methylation was observed in TT adults following intervention with the enzyme co-factor riboflavin [11].

We identified 246 differentially methylated CpG sites which were associated with both SBP and DBP (Fig. 3A). Only three CpG sites were identified to be associated with SBP, DBP and HTN, which may be due to the paucity of epigenome-wide methylation studies investigating HTN as an outcome. Three additional CpG sites were reported in five publications (Table S4), including cg19693031 annotated to the gene *TXNIP* [23, 28, 91–93]. Overexpression of TXNIP has been associated with increased oxidative stress and excessive ROS [94]. Seven studies report 1,391 DMRs to be significantly associated with various BP traits, with 56 of these common to both SBP and DBP (Fig. 3B). Eight DMRs were reported in more than one publication (Table S5).

Network analysis of genes annotated from CpG sites highlighted a protein cluster significantly enriched in biological processes associated with circadian rhythm (Fig. 4). Variations in BP occur naturally with circadian rhythm, and polymorphisms in genes associated with these proteins have been associated with both circadian phenotype and myocardial infarction [95–97]. Interestingly, functional analysis of all CpGs associated with BP traits highlights chromatin as the most significantly enriched cellular component (P_FDR_ = 1.59E-07) (Table S6) strengthening the evidence for the role of epigenetic modifications in HTN [97, 98].

Taken together, our results demonstrate that HTN is associated with accelerated epigenetic ageing indicating that EAA is higher in hypertensive individuals compared to normotensive individuals. The identification of robust biomarkers for accelerated epigenetic age in HTN patients may help cardiologists identify patients at greatest risk of complications and encourage lifestyle modifications such as exercise and targeted nutritional interventions. Further research is required to determine if EAA may be predictive of HTN outcomes or if measures to reduce EAA are useful in management of HTN.

## Strengths and Limitations

This review critically investigates the association between epigenetic age and HTN and importantly, is the first to employ a systematic approach to identify the studies subjected to meta-analysis to ensure a robust overview of current evidence. The analysis was conducted in line with PRISMA guidelines with defined inclusion and exclusion criteria. We have outlined a comprehensive summary using a combination of quantitative and qualitative evidence for the role of DNA methylation in HTN and BP. Limitations of this study include any fixed exposures that may influence epigenetic age, such as age, sex or race, which were not considered due to the small number of studies available for meta-analysis, however, all included studies bar one, cofounded for age and sex as appropriate [83, 99]. Studies included within the meta-analysis were considered to be sufficiently homogenous for analysis, however due to the exploratory nature of combining multiple epigenetic clocks studies, heterogeneity was introduced in the combined estimate limiting the generalisability of findings and we suggest caution in the interpretation of the pooled estimate broad summary of the overall association between EAA and HTN rather than a definitive result. A further limitation is that most studies included within the meta-analysis were conducted in the USA, while the remaining studies were conducted in other predominantly white Caucasian populations such as the UK and Australia. Although, 5/8 of included studies cofounded for ethnicity as a covariate, only one cohort included only African American participants (*n* = 227), introducing potential population bias and limiting generalisation of findings to non-European populations. The majority of studies assessed DNA methylation in blood due to sample accessibility with lack of readily available cardiac tissue studies remaining a constraint. DNA methylation exhibits tissue-specificity, influenced by leukocyte composition [100–105]. A recently defined cardiac-specific epigenetic clock indicated, however, that both blood and cardiac tissue reflect chronological age, suggesting that blood is a suitable proxy for cardiac epigenetic age studies [106, 107]. Moreover, the epigenetic clock algorithms applied in included studies were trained on multiple tissue types, including blood, and have been demonstrated robust performance across different tissues [16–19].

## Conclusion

This study is the first to demonstrate that HTN is associated with accelerated epigenetic ageing, by systematically evaluating the current evidence. There is need for further epigenome-wide approaches, and we recommend the use of clinically measured HTN over self-reported HTN in appropriately powered studies of epigenetic age to provide clarity on the relationship between environment, epigenome and HTN. The identification of robust biomarkers for accelerated epigenetic age in HTN patients may help clinicians identify patients at greatest risk of complications and encourage lifestyle modifications such as exercise and targeted nutritional interventions. In conclusion, accelerated epigenetic ageing as an underlying mechanism for hypertension holds much promise through the potential to impact development of novel therapeutic targets for HTN.

## Supplementary information

Supplementary Table S1

Supplementary Table S2

Supplementary Table S3

Supplementary Table S4

Supplementary Table S5

Supplementary Table S6

Supplementary Table S7

Supplementary Figure S1

Supplementary Figure S2

Supplementary Figure S3

Supplementary Figures Legend

## Data Availability

All data used within this study were obtained from previously published articles and publicly available sources. Full details of data sources, including citations are available in the main text tables and supplementary materials. Readers may access the original datasets by referring to the cited publications.
