# Supplementary material for: Accelerated epigenetic age in hypertension: a systematic review and meta-analysis
Source: Hypertens Res. 2026 Jan 9;49(4):1265–303. doi: 10.1038/s41440-025-02470-y (PMC13050651; doi:10.1038/s41440-025-02470-y)
Supplement: Supplementary file 1 — Supplementary Table S1 [file 41440_2025_2470_MOESM1_ESM.docx]

Table S1: PICOS (population, intervention, comparison, outcome and study design) criteria for inclusion and exclusion of studies.

| Parameter | Criteria |
| --- | --- |
| Participants | Adults aged 18 years or older where BP has been measured. |
| Exposure | Hypertension/Blood pressure |
| Comparison | Measured of DNA methylation (global, gene-specific, genome-wide, epigenetic age). |
| Outcome | Increased epigenetic age acceleration, altered DNA methylation at specific sites. |
| Study design | RCT, Observational studies including cross-sectional, case-control and cohort studies. |
