# Supplementary material for: Accelerated epigenetic age in hypertension: a systematic review and meta-analysis
Source: Hypertens Res. 2026 Jan 9;49(4):1265–303. doi: 10.1038/s41440-025-02470-y (PMC13050651; doi:10.1038/s41440-025-02470-y)
Supplement: Supplementary file 2 — Supplementary Table S2 [file 41440_2025_2470_MOESM2_ESM.docx]

Table S2: Systematic search strategy

| Systematic literature search was carried out in six databases (Medline, Scopus, Embase, Web of Science, CINHAL plus and Cochrane library) to identify publications describing the association between DNA methylation and blood pressure and/or hypertension in adult humans. The search strategy is described below: |
| --- |
| 1. exp Epigenomics/  2. exp DNA Methylation/  3. exp 5-Methylcytosine/  4. exp S-Adenosylmethionine/  5. exp Methylation/  6. ("DNA methylation" or "Epigenomewide Methylation" or "genomewide methylation" or "global DNA methylation" or "gene-specific DNA methylation" or "5-Methylcytosine" or "Epigenome wide" or "epigenome-wide" or epigen* or epigenetics or epigenomewide or methylat* or demethylat* or CpG or 450K or Methyl450K or Methylation450 or beadchip or "bead chip" or 850K or EWAS or "genome-wide methylation" or "illumina 450K" or "illumina 850K" or MethylationEPIC or "Infinium Methylation EPIC" or "5-hydroxymethylcytosine").af.  7. exp Hypertension/  8. exp Blood Pressure/  9. ("High Blood Pressure" or Hypertension or "systolic pressure" or hyperten* or "elevated blood pressure" or htn or "blood pressure" or "diastolic pressure").af  10. 1 or 2 or 3 or 5 or 6  11. 7 or 8 or 9  12. 10 and 11  13. Limit 12 to (English language and full text and humans) |
