# Supplementary material for: Accelerated epigenetic age in hypertension: a systematic review and meta-analysis
Source: Hypertens Res. 2026 Jan 9;49(4):1265–303. doi: 10.1038/s41440-025-02470-y (PMC13050651; doi:10.1038/s41440-025-02470-y)
Supplement: Supplementary file 3 — Supplementary Table S3 [file 41440_2025_2470_MOESM3_ESM.docx]

Table S3: Risk of bias assessment of observational studies with associations between DNA methylation and blood pressure and/or HTN.

|  | **Case Control studies** | |  |
| --- | --- | --- | --- |
| Study | Subject selection (Max. 4★) | Study Comparability (Max. 2★) | Ascertainment of exposure (Max. 3★) |
| Abula *et al*., 2023 | ★★★ |  | ★★ |
| Amenyah *et al*., 2020 | ★★★ | ★★ | ★★ |
| Amenyah *et al*., 2021 | ★★★ | ★★ | ★★ |
| Baccarelli *et al*., 2015 | ★ |  | ★★★ |
| Bai *et al*., 2022 | ★★★ | ★★ | ★★★ |
| Bao *et al*., 2018 | ★★ | ★★ | ★★★ |
| Bayoumy *et al*., 2017 | ★★ |  | ★★★ |
| Bi *et al*., 2024 | ★★★ | ★★ | ★★ |
| Bushueva *et al*., 2021 | ★★ | ★ | ★★ |
| Castellano-Castillo *et al*., 2018 | ★★ | ★ | ★★★ |
| Castellano-Castillo *et al*., 2019 | ★★ | ★ | ★★★ |
| Das *et al*., 2016 | ★★★ | ★ | ★★★ |
| Deng *et al*., 2018 | ★★ | ★ | ★★★ |
| El Alami *et al*., 2022 | ★★ |  | ★★★ |
| Fan *et al*., 2015^a^ | ★★★ |  | ★★★ |
| Fan *et al*., 2015^b^ | ★★★ | ★ | ★★★ |
| Fan *et al*., 2017 | ★★★ | ★ | ★★★ |
| Ferrari *et al*., 2019 | ★★★ | ★ | ★★ |
| Giannakopoulou *et al*., 2017 | ★★ | ★ | ★★★ |
| Guo *et al*., 2020 | ★★ | ★ | ★★★ |
| Gu *et al*., 2016 | ★★ | ★ | ★★★ |
| Jin *et al*., 2018 | ★★ |  | ★★★ |
| Jin *et al*., 2022 | ★★ | ★ | ★★★ |
| Koochankkhani *et al*., 2021 | ★★★ | ★ | ★★ |
| Krishnan *et al*., 2017 | ★★ |  | ★★★ |
| Li *et al.,* 2022 | ★★★ | ★★ | ★★★ |
| Li *et al*., 2023 | ★★★ | ★ | ★★★ |
| Lin *et al*., 2017 | ★★★ | ★★ | ★★★ |
| Li-Tempel *et al*., 2016 | ★★ | ★ | ★★★ |
| Lopez-Legarrea *et al*., 2013 | ★★ |  | ★★★ |
| Maghbooli *et al*., 2014 | ★★ | ★ | ★★ |
| Maghbooli *et al*., 2015 | ★★ | ★ | ★★ |
| Mao *et al*., 2016 | ★★★ | ★★ | ★★★ |
| Mao *et al*., 2017^a^ | ★★★ | ★★ | ★★★ |
| Mao *et al*., 2017^b^ | ★★★ | ★ | ★★★ |
| Mo *et al*., 2020 | ★★★ | ★★ | ★★★ |
| Osum *et al*., 2023 | ★★ |  | ★★ |
| Peng *et al*., 2014 | ★★ | ★★ | ★★★ |
| Si *et al*., 2021 | ★★★ | ★★ | ★★★ |
| Smolarek *et al*., 2010 | ★★ |  | ★★★ |
| Wang *et al*., 2019^a^ | ★★★ | ★ | ★★★ |
| Wang *et al*., 2019^b^ | ★★★ |  | ★★★ |
| Wei *et al*., 2014 | ★★ | ★★ | ★★ |
| Wei *et al*., 2016 | ★★ | ★ | ★★★ |
| Wu *et al*., 2017 | ★★ | ★ | ★★★ |
| Xu *et al*., 2019^a^ | ★★★ |  | ★★★ |
| Xu *et al*., 2019^b^ | ★★★ | ★★ | ★★★ |
| Xu *et al*., 2020^a^ | ★★★ | ★ | ★★ |
| Xu *et al*., 2020^b^ | ★★★ | ★ | ★★★ |
| Xu *et al*., 2021 | ★★ | ★★ | ★★★ |
| Yadav *et al*., 2021 | ★★ | ★ | ★★★ |
| Zhang *et al*., 2013^a^ | ★★★ | ★ | ★★★ |
| Zhong *et al*., 2016 | ★★★ | ★★ | ★★★ |
|  | **Cohort studies** | |  |
| Study | Subject selection (Max. 4★) | Study Comparability (Max. 2★) | Ascertainment of exposure (Max. 3★) |
| Breitling *et al*., 2012 | ★★ |  | ★★★ |
| Ciantar *et al*., 2024 | ★★ | ★★ | ★★ |
| Chen *et al*., 2024 | ★★★ | ★★ | ★★ |
| Chiu *et al*., 2023 | ★★★ | ★★ | ★★★ |
| Gao *et al*., 2018 | ★★★ | ★ | ★★★ |
| Guay *et al*., 2014 | ★★ | ★ | ★★★ |
| Huang *et al*., 2016 | ★★★ | ★ | ★★★ |
| Li *et al*., 2019 | ★★★ | ★★ | ★★ |
| Li *et al*., 2020 | ★★★ | ★★ | ★★★ |
| Marotta *et al*., 2021 | ★★★ | ★★ | ★ |
| McCartney *et al*., 2018 | ★★★ | ★★ | ★★★ |
| Meng *et al*., 2017 | ★★★ | ★ | ★★ |
| Mens *et al*., 2020 | ★★ | ★★ | ★★★ |
| Qin *et al*., 2021 | ★★★ | ★★ | ★★ |
| Robinson *et al*., 2020 | ★★★ | ★★ | ★★★ |
| Roetker *et al*., 2018 | ★★★ | ★★ | ★★★ |
| Shi *et al*., 2023 | ★★ | ★★ | ★★ |
| Smith *et al*., 2019 | ★★★ | ★★ | ★★★ |
| Sprague *et al*., 2022 | ★★ | ★★ | ★★ |
| Zhang *et al*., 2022^b^ | ★★★ | ★★ | ★★★ |
|  | **Cross sectional studies** | |  |
| Study | Subject selection (Max. 5★) | Study Comparability (Max. 1★) | Ascertainment of exposure (Max. 3★) |
| Alexeeff *et al*., 2013 | ★★★ | ★ | ★★★ |
| Ali *et al*., 2021 | ★★ |  | ★★★ |
| Ammous *et al*., 2021 | ★★★ | ★ | ★★★ |
| Arpon *et al*., 2019 | ★★★ |  | ★★★ |
| Baccarelli *et al*., 2010 | ★★ | ★ | ★★★ |
| Bellavia *et al*., 2013 | ★★ |  | ★★★ |
| Boström *et al*., 2016 | ★★ | ★ | ★★★ |
| Burghardt *et al*., 2012 | ★★ | ★ | ★★★ |
| Carraro *et al*., 2016^a^ | ★★★ |  | ★★★ |
| Carraro *et al*., 2016^b^ | ★★★ | ★ | ★★★ |
| Caspers *et al*., 2019 | ★★ | ★ | ★★ |
| Chen *et al*., 2014 | ★★★ |  | ★★★ |
| Chen *et al*., 2020 | ★★★ | ★ | ★★ |
| Chen *et al*., 2023 | ★★★ | ★ | ★★★ |
| Childebayeva *et al*., 2019 | ★★ | ★ | ★★★ |
| Chilunga *et al*., 2021 | ★★★★ | ★ | ★★★ |
| Cordero *et al*., 2011 | ★★ |  | ★★★ |
| Corsi *et al*., 2020 | ★★★ | ★ | ★★★ |
| Cortese *et al*., 2016 | ★★★ |  | ★★ |
| Cronjé *et al*., 2020 | ★★★ | ★ | ★★★ |
| da Silva Rodrigues *et al.,* 2024 | ★★★ | ★ | ★★★ |
| Diniz *et al*., 2021 | ★★ |  | ★★★ |
| Drake *et al*., 2012 | ★★★ | ★ | ★★★ |
| Dugue *et al*., 2018 | ★★★ | ★ | ★★★ |
| Fernandez-Sanles *et al*., 2018 | ★★★ | ★ | ★★★ |
| Friso *et al*., 2008 | ★★★ |  | ★★ |
| Föhr *et al*., 2024 | ★★ | ★ | ★★★ |
| Gao *et al*., 2016 | ★★ | ★ | ★★ |
| Hernández-Cordero *et al*., 2022 | ★★ | ★ | ★★ |
| Hong *et al*., 2023 | ★★★ | ★ | ★★★ |
| Horvath *et al*., 2016 | ★★★★ | ★ | ★★★ |
| Horvath *et al*., 2022 | ★★ | ★ | ★★★ |
| Hossain *et al*., 2017 | ★★★ | ★ | ★★★ |
| Huan *et al*., 2019 | ★★★ | ★ | ★★★ |
| Irvin *et al*., 2018 | ★★★ | ★ | ★★★ |
| Jiang *et al*., 2022 | ★★★ | ★ | ★★★ |
| Jiraboonsri *et al*., 2023 | ★★ |  | ★★ |
| Kato *et al*., 2015 | ★★★★ | ★ | ★★★ |
| Kazmi *et al*., 2020 | ★★★★ | ★ | ★★ |
| Kho *et al*., 2020 | ★★★ | ★ | ★★ |
| Kidambi *et al*., 2021 | ★★ | ★ | ★★★ |
| Kim *et al*., 2010 | ★★ | ★★ | ★★★ |
| Kou *et al*., 2023 | ★★★ | ★ | ★★★ |
| Kresovich *et al.,* 2023 | ★★ | ★ | ★★★ |
| Kucher *et al*., 2017 | ★ | ★ | ★★★ |
| Lee *et al.,* 2024 | ★★★ | ★ | ★★★ |
| Levine *et al*., 2018 | ★★★ | ★★ | ★★★ |
| Li *et al*., 2021 | ★★ | ★ | ★★★ |
| Lim *et al*., 2017 | ★★★ | ★ | ★★★ |
| Lind *et al*., 2018 | ★★ | ★ | ★★★ |
| Lin *et al*., 2016 | ★★ | ★ | ★★★ |
| Lin *et al.,* 2023 | ★★ | ★ | ★★ |
| Lo *et al*., 2022 | ★★ | ★ | ★★★ |
| Lu *et al*., 2019 | ★★★ | ★ | ★★★ |
| Lu *et al*., 2020 | ★★★ | ★ | ★★★ |
| Luttmer *et al*., 2013 | ★★ | ★ | ★★★ |
| Macías- Gonzalez *et al.,* 2018 | ★★★ | ★ | ★★★ |
| Mansouri *et al*., 2022 | ★★ | ★ | ★★★ |
| Marinello *et al*., 2024 | ★★ |  | ★★ |
| Marioni *et al*., 2015 | ★★★★ | ★ | ★★★ |
| Marques-Rocha *et al*., 2016 | ★ | ★ | ★★★ |
| Milagro *et al*., 2011 | ★ | ★ | ★★★ |
| Milagro *et al*., 2012 | ★ | ★ | ★★★ |
| Nuotio *et al*., 2020 | ★★★ | ★ | ★★★ |
| Pan *et al*., 2024 | ★★ | ★ | ★★★ |
| Perez *et al*., 2022 | ★★ | ★ | ★★★ |
| Philibert *et al,* 2024 | ★★ | ★ | ★★ |
| Portilla-Fernandez *et al.,* 2019 | ★★★ | ★ | ★★★ |
| Pottinger *et al.,* 2021 | ★★★ | ★ | ★★★ |
| Quach *et al*., 2017 | ★★★ | ★ | ★★★ |
| Ramos-Lopez *et al*., 2018 | ★★ | ★ | ★★★ |
| Richard *et al*., 2017 | ★★★★ | ★ | ★★★ |
| Roberts *et al*., 2021 | ★★★ | ★ | ★★★ |
| Roberts *et al*., 2022 | ★★ | ★ | ★★★ |
| Tamman *et al*., 2019 | ★★ | ★ | ★★★ |
| Tang *et al*., 2023 | ★★ | ★ | ★★ |
| Thongsroy *et al*., 2017 | ★★ | ★ | ★★★ |
| Thongsroy *et al*., 2022 | ★★ | ★ | ★★★ |
| Turcot *et al*., 2012 | ★★ | ★ | ★★★ |
| Turcot *et al*., 2013 | ★★ | ★ | ★★★ |
| Wan *et al*., 2024 | ★★ | ★ | ★★★ |
| Wang *et al*., 2014 | ★★ |  | ★★★ |
| Wang *et al.,* 2023 | ★★★ | ★ | ★★ |
| Wang *et al*., 2024 | ★★★ | ★ | ★★★ |
| Wan Omar *et al*., 2019 | ★★ |  | ★★★ |
| Wu *et al*., 2022 | ★★ | ★ | ★★★ |
| Xia *et al.,* 2023 | ★★ | ★ | ★★★ |
| Xu *et al*., 2024 | ★★ | ★ | ★★ |
| Xiao *et al*., 2022 | ★★★ | ★ | ★★★ |
| Yang *et al*., 2016 | ★★ |  | ★★★ |
| Yao *et al*., 2024 | ★★ | ★ | ★★★ |
| Zhang *et al*., 2013^b^ | ★★ | ★ | ★★★ |
| Zhang *et al*., 2022^a^ | ★★★ | ★ | ★★★ |

Risk of bias assessment of observational studies were carried out using the Newcastle-Ottawa quality assessment scale. A study can be awarded a maximum of one star for each numbered item within the selection and outcome categories. A maximum of two stars can is given for comparability.
