# Supplementary material for: Accelerated epigenetic age in hypertension: a systematic review and meta-analysis
Source: Hypertens Res. 2026 Jan 9;49(4):1265–303. doi: 10.1038/s41440-025-02470-y (PMC13050651; doi:10.1038/s41440-025-02470-y)
Supplement: Supplementary file 4 — Supplementary Table S4 [file 41440_2025_2470_MOESM4_ESM.docx]

Table S4: CpG sites reported in more than one publication and corresponding BP outcomes.

| CpG site | No. studies reported | Annotated gene | Outcomes associated | References |
| --- | --- | --- | --- | --- |
| *Overall* |  |  |  |  |
| cg19693031 | 5 | *TXNIP* | SBP, DBP | Hong *et al*., 2023; Zhang *et al*., 2022b; Kho *et al*., 2020; Nuotio *et al*., 2020; Richard *et al*., 2017 |
| cg18120259 | 5 | *LOC100132354* | SBP, DBP, HTN | Hong *et al*., 2023; Zhang *et al*., 2022b; Kazmi *et al*., 2020; Kho *et al*., 2020; Richard *et al*., 2017 |
| cg00574958 | 5 | *CPT1A* | SBP, DBP, HTN | Hong *et al*., 2023; Kho *et al*., 2020; Richard *et al*., 2017; Das *et al*., 2016; Lee *et al*., 2024 |
| cg06690548 | 3 | *SLC7A11* | SBP, DBP | Hong *et al*., 2023; Zhang *et al*., 2022b; Richard *et al*., 2017 |
| cg14476101 | 3 | *PHGDH* | SBP, DBP | Hong *et al*., 2023; Zhang *et al*., 2022b; Richard *et al*., 2017 |
| cg16246545 | 3 | *PHGDH* | SBP, DBP | Hong *et al*., 2023; Zhang *et al*., 2022b; Richard *et al*., 2017 |
| cg10833066 | 3 | *FAM109A* | SBP, DBP | Mo *et al*., 2020; Huan *et al*., 2019; Kato *et al*., 2015 |
| cg17058475 | 3 | *CPT1A* | SBP, DBP, HTN | Hong *et al*., 2023; Richard *et al*., 2017; Das *et al*., 2016 |
| cg00533891 | 2 | *ZMIZ1* | SBP, DBP | Zhang *et al*., 2022b; Richard *et al*., 2017 |
| cg00730441 | 2 | *TBX2* | SBP, MAP | Mo *et al*., 2020; Kato *et al*., 2015 |
| cg00805360 | 2 | *ADAM8* | SBP, DBP | Hong *et al*., 2023; Richard *et al*., 2017 |
| cg02108620 | 2 | *ULK4* | DBP, PP | Mo *et al*., 2020; Kato *et al*., 2015 |
| cg02711608 | 2 | *SLC1A5* | SBP, DBP | Zhang *et al*., 2022b; Richard *et al*., 2017 |
| cg02976539 | 2 | *SLC9A3R1* | SBP, DBP | Hong *et al*., 2023; Richard *et al*., 2017 |
| cg03493300 | 2 | *CNNM2* | SBP, DBP | Mo *et al*., 2020; Huan *et al*., 2019 |
| cg04427651 | 2 | *ZDHHC18* | SBP, DBP | Hong *et al*., 2023; Richard *et al*., 2017 |
| cg05228408 | 2 | *MTHFR; CLCN6* | SBP, DBP, MAP | Lin *et al*., 2016; Kato *et al*., 2015 |
| cg05632420 | 2 | *MKLN1; FLJ43663* | SBP, DBP | Hong *et al*., 2023; Richard *et al*., 2017 |
| cg06330618 | 2 | *FES* | SBP, DBP | Mo *et al*., 2020; Kato *et al*., 2015 |
| cg06688763 | 2 | *CDK6* | SBP, DBP | Hong *et al*., 2023; Richard *et al*., 2017 |
| cg06826457 | 2 | *chr12:12867669* | SBP, DBP | Hong *et al*., 2023; Richard *et al*., 2017 |
| cg09680149 | 2 | *SCNN1A* | SBP, DBP | Hong *et al*., 2023; Richard *et al*., 2017 |
| cg10751070 | 2 | *TBC1D12* | SBP, DBP, PP | Huan *et al*., 2019; Kato *et al*., 2015 |
| cg11376147 | 2 | *SLC43A1* | SBP, DBP | Hong *et al*., 2023; Richard *et al*., 2017 |
| cg12593793 | 2 | *LMNA* | SBP, DBP | Hong *et al*., 2023; Richard *et al*., 2017 |
| cg14741228 | 2 | *FAM60A; FLJ13224* | SBP, DBP | Hong *et al*., 2023; Richard *et al*., 2017 |
| cg15114651 | 2 | *SLC1A5* | SBP, DBP | Hong *et al*., 2023; Richard *et al*., 2017 |
| cg15616915 | 2 | *CTDSP1; MIR26B* | SBP, DBP | Mens *et al*., 2020; Richard *et al*., 2017 |
| cg15741354 | 2 | *PRAG1* | SBP, DBP | Mo *et al*., 2020; Huan *et al*., 2019 |
| cg15920975 | 2 | *NEAT1* | SBP, DBP | Hong *et al*., 2023; Richard *et al*., 2017 |
| cg17061862 | 2 | *chr11:9590431* | SBP, DBP | Zhang *et al*., 2022b; Richard *et al*., 2017 |
| cg17443080 | 2 | *EGFL7* | SBP, DBP | Hong *et al*., 2023; Richard *et al*., 2017 |
| cg18824549 | 2 | *FAM117A* | SBP, DBP | Hong *et al*., 2023; Richard *et al*., 2017 |
| cg18933331 | 2 | *ch1:110186418* | SBP, DBP | Hong *et al*., 2023; Richard *et al*., 2017 |
| cg19266329 | 2 | *NBPF8: NBPF12* | SBP, DBP | Hong *et al*., 2023; Richard *et al*., 2017 |
| cg20734569 | 2 | *SPINK8* | SBP, DBP | Mo *et al*., 2020; Huan *et al*., 2019 |
| cg21033440 | 2 | *SIPA1* | SBP, DBP | Mo *et al*., 2020; Huan *et al*., 2019 |
| cg21618521 | 2 | *B3GALT4* | SBP, DBP | Hong *et al*., 2023; Richard *et al*., 2017 |
| cg22052056 | 2 | *DNMT3B* | SBP, DBP | Hong *et al*., 2023; Richard *et al*., 2017 |
| cg22213445 | 2 | *RAB7A* | SBP, DBP | Hong *et al*., 2023; Richard *et al*., 2017 |
| cg22304262 | 2 | *SLC1A5* | SBP, DBP | Zhang *et al*., 2022b; Richard *et al*., 2017 |
| cg22959409 | 2 | *VPS37B* | SBP, DBP | Hong *et al*., 2023; Richard *et al*., 2017 |
| cg23999170 | 2 | *TSPAN2* | SBP, DBP | Hong *et al*., 2023; Richard *et al*., 2017 |
| cg24468199 | 2 | *SIPA1* | SBP, DBP | Mo *et al*., 2020; Huan *et al*., 2019 |
| cg25203007 | 2 | *GALE* | SBP, DBP | Hong *et al*., 2023; Richard *et al*., 2017 |

*CpG sites ranked by the number of studies in which they are reported.*
