# Supplementary material for: Accelerated epigenetic age in hypertension: a systematic review and meta-analysis
Source: Hypertens Res. 2026 Jan 9;49(4):1265–303. doi: 10.1038/s41440-025-02470-y (PMC13050651; doi:10.1038/s41440-025-02470-y)
Supplement: Supplementary file 5 — Supplementary Table S5 [file 41440_2025_2470_MOESM5_ESM.docx]

Table S5: Genomic locations of differentially methylated regions (DMRs) which are reported in more than one publication and genes annotated from separate DMRs with corresponding BP outcomes.

|  | No. studies reported | Overlapping DMRs* | Outcomes associated | References |
| --- | --- | --- | --- | --- |
| Gene |  |  |  |  |
| *GLIPR1L2* | 2 | chr12:75784541-75785295;  chr12:75784855-75785295 | SBP, HTN | Kazmi *et al*., 2020; Xiao *et al*., 2022 |
| *LOC100996842:MPDU1* | 2 | chr17:7486550-7486725; chr17:7486551-7487249 | SBP | Roberts et al., 2022; Kazmi *et al*., 2020 |
| *MPRIP* | 2 | chr17:17062144-17062244 | SBP, DBP, MAP | Roberts *et al*., 2022; Pan *et al*., 2024 |
| *COLGALT1* | 2 | chr19:17688057-17688109 | SBP, DBP, MAP | Roberts *et al*., 2022; Pan *et al*., 2024 |
| *DVL3* | 2 | chr3:183887775-183887926;  chr3:183887905-183888477 | DBP | Kazmi *et al*., 2020; Pan *et al*., 2024 |
| *N/A* | 2 | chr4:70442120-70442358 | SBP, DBP | Roberts *et al*., 2022; Pan *et al*., 2024 |
| *HOPX* | 2 | chr4:57547347-57547872;  chr4:57547347-57548290 | DBP, HTN | Kazmi *et al*., 2020; Xiao *et al*., 2022 |
| *N/A* | 2 | chr6:31650735-31651070;  chr6:31650735-31651362 | SBP, HTN | Kazmi *et al*., 2020; Xiao *et al*., 2022 |
| Gene annotated from separate DMRs | No. studies reported | Separate DMRs | Outcomes associated |  |
| *NOTCH1* | 2 | chr9:139410088-139410088;  chr9:139410711-139410905 | DBP, MAP | Roberts et al., 2022; Kazmi *et al*., 2020 |
| *CASZ1* | 2 | chr1:10797871-10797871;  chr1:10811726-10811764 | SBP, DBP, MAP | Roberts *et al*., 2022; Pan *et al*., 2024 |
| *CACNA1C* | 2 | chr12:2412228-2412311;  chr12:2800055-2800055 | SBP, MAP | Kazmi et al., 2020; Pan et al., 2024 |
| *ADPRHL1* | 2 | chr13:114081075-114081250;  chr13:114103452-114103796 | DBP | Kazmi et al., 2020; Pan et al., 2024 |
| *TSHZ1* | 2 | chr18:72922610-72923257;  chr18:72922610-72923014;  chr18:72956570-72956683 | SBP | Kazmi et al., 2020; Pan et al., 2024 |
| *GNG7* | 2 | chr19:2543602-2544100;  chr19:2611689-2611757 | SBP, DBP, MAP | Kazmi et al., 2020; Pan et al., 2024 |
| *AGAP1* | 2 | chr2:236540006-236540072;  chr2:236579007-236580208 | SBP | Kazmi et al., 2020; Pan et al., 2024 |
| *RHOB* | 2 | chr2:20646664-20646880;  chr2:20648179-20648223 | DBP, MAP | Kazmi et al., 2020; Pan et al., 2024 |
| *WDR27* | 2 | chr6:170047852-170048424;  chr6:169965078-169965142 | SBP, DBP, MAP | Kazmi et al., 2020; Pan et al., 2024 |
| *VAV2* | 2 | chr9:136698948-136699122;  chr9:136677064-136677533 | SBP, DBP | Kazmi et al., 2020; Pan et al., 2024 |
| *PRDM16* | 2 | chr1:3155180-3155210;  chr1:3001002-3001128 | DBP | Kazmi et al., 2020; Pan et al., 2024 |

*Differentially methylated regions (DMRs) which overlap and are recorded in >1 publication.
