# Supplementary material for: Accelerated epigenetic age in hypertension: a systematic review and meta-analysis
Source: Hypertens Res. 2026 Jan 9;49(4):1265–303. doi: 10.1038/s41440-025-02470-y (PMC13050651; doi:10.1038/s41440-025-02470-y)
Supplement: Supplementary file 6 — Supplementary Table S6 [file 41440_2025_2470_MOESM6_ESM.docx]

Table S6: FDR Significant enriched pathways in CpG sites associated with BP traits

| Pathway | Count | % | Fold Enrichment | P-Value | FDR |
| --- | --- | --- | --- | --- | --- |
| Disease Terms |  |  |  |  |  |
| Systemic Lupus Erythematosus | 47 | 9.92 | 5.40 | 5.91E-21 | 1.84E-17 |
| Schizophrenia | 28 | 5.91 | 2.96 | 9.00E-07 | 0.001 |
| Hypertension | 31 | 6.54 | 2.65 | 2.16E-06 | 0.002 |
| Systolic blood pressure | 7 | 1.48 | 13.84 | 6.78E-06 | 0.005 |
| Psoriasis | 15 | 3.16 | 4.20 | 1.27E-05 | 0.008 |
| Spontaneous Abortion | 13 | 2.74 | 4.48 | 3.14E-05 | 0.016 |
| HIV-1 Control | 6 | 1.27 | 14.09 | 4.39E-05 | 0.020 |
| Cellular component |  |  |  |  |  |
| Chromatin | 46 | 9.70 | 1.82 | 1.06E-05 | 0.005 |
| Cytoplasm | 157 | 33.12 | 1.27 | 3.22E-05 | 0.007 |
| Endosome membrane | 17 | 3.59 | 2.90 | 0.0003 | 0.029 |
| Nucleoplasm | 110 | 23.21 | 1.34 | 0.0006 | 0.035 |
| Cytosol | 144 | 30.38 | 1.26 | 0.0004 | 0.037 |
| Lumenal side of endoplasmic reticulum membrane | 6 | 1.27 | 2.38 | 0.0006 | 0.040 |
| Nucleus | 155 | 32.70 | 2.84 | 0.0006 | 0.040 |

Functional analysis conducted using DAVID bioinformatics resource for genes annotated to CpG sites associated with BP traits.
