# Supplementary material for: Accelerated epigenetic age in hypertension: a systematic review and meta-analysis
Source: Hypertens Res. 2026 Jan 9;49(4):1265–303. doi: 10.1038/s41440-025-02470-y (PMC13050651; doi:10.1038/s41440-025-02470-y)
Supplement: Supplementary file 7 — Supplementary Table S7 [file 41440_2025_2470_MOESM7_ESM.docx]

Table S7: FDR Significant enriched pathways in DMRs associated with BP traits

| Pathway | Count | % | Fold Enrichment | P-Value | FDR |
| --- | --- | --- | --- | --- | --- |
| Biological processes |  |  |  |  |  |
| Regulation of transcription by RNA polymerase II | 73 | 8.41 | 1.94 | 9.17E-08 | 0.0003 |
| Cartilage development | 15 | 1.73 | 5.78 | 2.14E-07 | 0.0004 |
| Intracellular signal transduction | 40 | 4.60 | 2.16 | 7.43E-06 | 0.0085 |
| Molecular Function |  |  |  |  |  |
| Protein binding | 621 | 71.54 | 1.11 | 9.81E-08 | 0.0001 |
| RNA polymerase II-specific DNA-binding transcription factor activity | 88 | 10.14 | 1.72 | 7.32E-07 | 0.0003 |
| Metal ion binding | 169 | 19.47 | 1.43 | 7.42E-07 | 0.0003 |
| Zinc ion binding | 126 | 14.52 | 1.41 | 4.63E-05 | 0.011 |
| Sequence-specific double-stranded DNA binding | 44 | 5.07 | 1.90 | 7.23E-05 | 0.013 |
| Identical protein binding | 103 | 11.87 | 1.42 | 0.00022 | 0.034 |
| Cellular components |  |  |  |  |  |
| Chromatin | 88 | 10.13 | 2.04 | 2.75E-10 | 1.59E-07 |
| Nucleoplasm | 212 | 24.42 | 1.36 | 8.43E-07 | 0.0002 |
| Post synaptic density | 26 | 3.00 | 2.66 | 1.73E-05 | 0.0033 |
| Cell surface | 45 | 5.18 | 1.79 | 0.00024 | 0.032 |
| Focal adhesion | 33 | 3.80 | 2.00 | 0.00028 | 0.032 |
| Synapse | 37 | 4.26 | 1.84 | 0.00057 | 0.049 |
| Axon | 28 | 3.23 | 2.06 | 0.00059 | 0.049 |

Functional analysis conducted using DAVID bioinformatics resource for genes annotated to DMRs associated with BP traits.
