# Supplementary figures and images for: Accelerated epigenetic age in hypertension: a systematic review and meta-analysis
Source: Hypertens Res. 2026 Jan 9;49(4):1265–303. doi: 10.1038/s41440-025-02470-y (PMC13050651; doi:10.1038/s41440-025-02470-y)

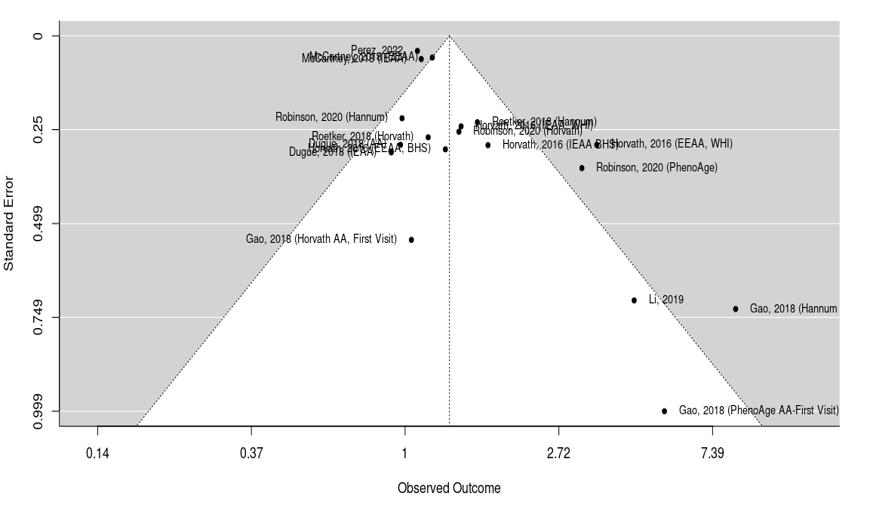

Supplement: Supplementary file 8 — Supplementary Figure S1 [file 41440_2025_2470_MOESM8_ESM.tif]

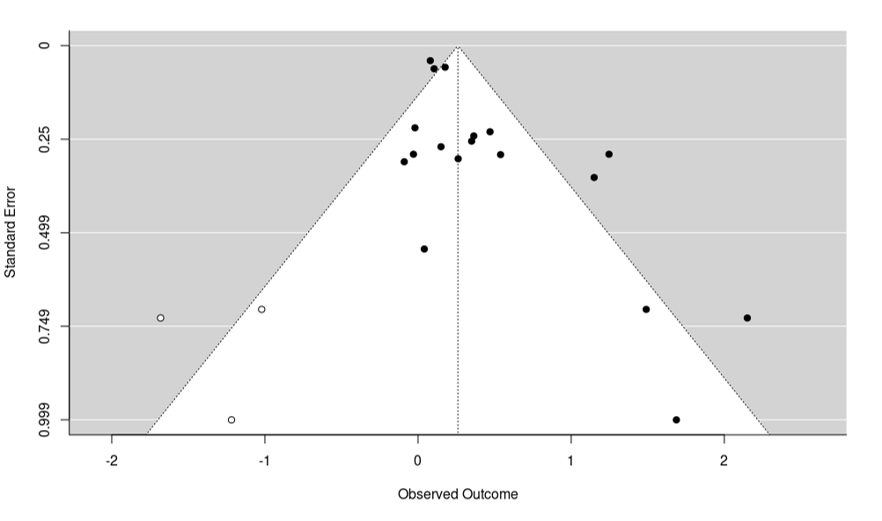

Supplement: Supplementary file 9 — Supplementary Figure S2 [file 41440_2025_2470_MOESM9_ESM.tif]

## Meta-analysis estimates – Given study is omitted

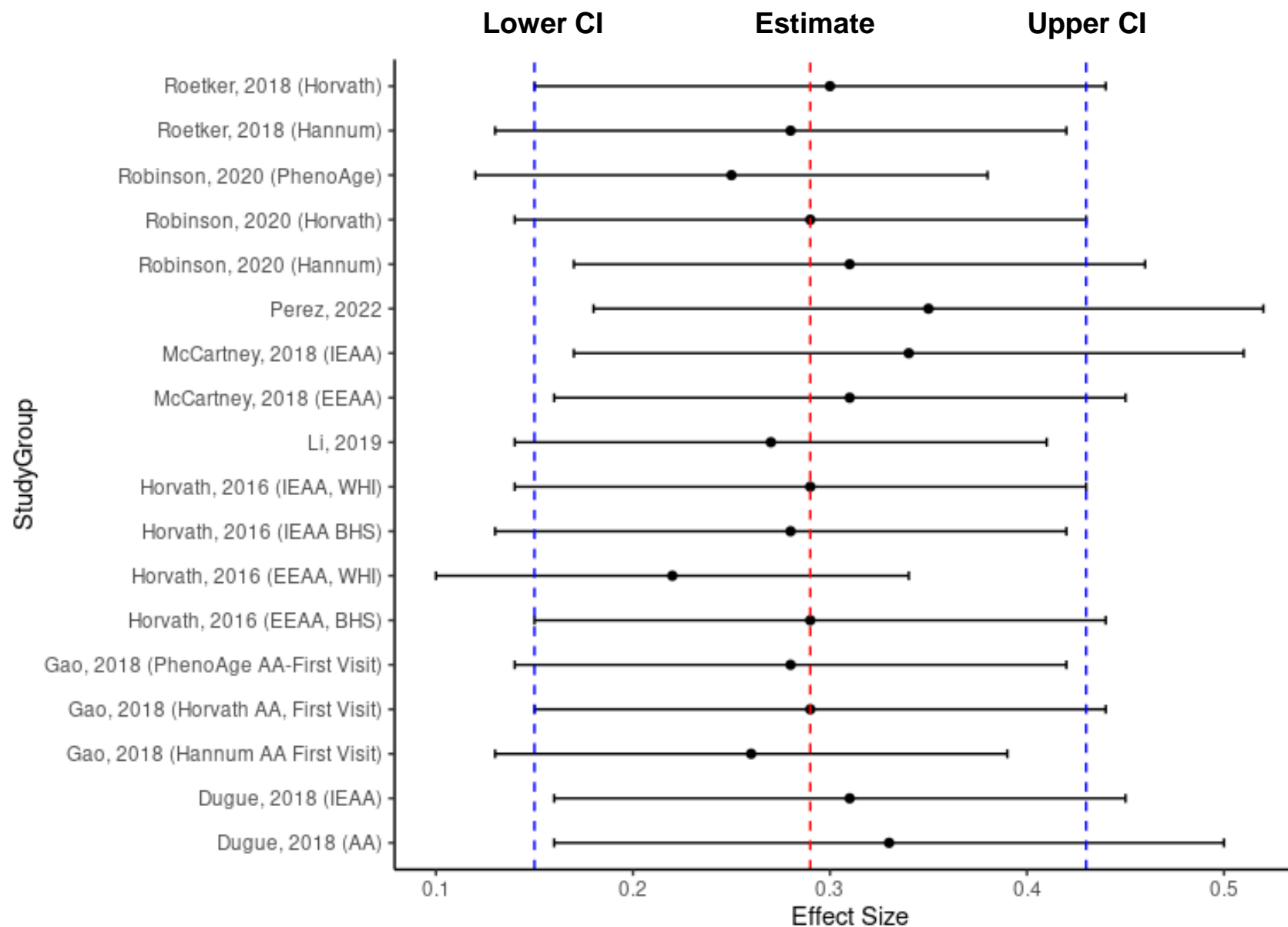

Supplement: Supplementary file 10 — Supplementary Figure S3 [file 41440_2025_2470_MOESM10_ESM.pdf]
