# Supplementary material for: Accelerated epigenetic age in hypertension: a systematic review and meta-analysis
Source: Hypertens Res. 2026 Jan 9;49(4):1265–303. doi: 10.1038/s41440-025-02470-y (PMC13050651; doi:10.1038/s41440-025-02470-y)
Supplement: Supplementary file 11 — Supplementary Figures Legend [file 41440_2025_2470_MOESM11_ESM.docx]

Figure S1 Funnel plot of the meta-analysis of published studies. Each plotted point represents the standard error and observed outcome of association between hypertension and epigenetic age acceleration. The white triangle represents the region where 95% of the data points should lie in absence of publication bias. Vertical line represents the average observed outcome found within the meta-analysis.

Figure S2 Funnel plot adjusted using the trim-and-fill method. Black circles: Included comparisons; White circles: inputted comparisons using the trim-and-fill method. The white triangle represents the region where 95% of the data points should lie in absence of publication bias. Vertical line represents the average observed outcome found within the meta-analysis.

Figure S3 Sensitivity analysis outlining meta-analysis estimates where given study name is omitted. Original meta-analysis estimation is given as the red dotted line while the original 95% confidence interval is denoted as blue dotted lines.
